# Astroglial β-Arrestin1-mediated Nuclear Signaling Regulates the Expansion of Neural Precursor Cells in Adult Hippocampus

**DOI:** 10.1038/srep15506

**Published:** 2015-10-26

**Authors:** Yezheng Tao, Li Ma, Zhaohui Liao, Qiumin Le, Jialing Yu, Xing Liu, Haohong Li, Yuejun Chen, Ping Zheng, Zhengang Yang, Lan Ma

**Affiliations:** 1 The State Key Laboratory of Medical Neurobiology, School of Basic Medical Sciences, the Institutes of Brain Science, and the Collaborative Innovation Center for Brain Science, Fudan University, Shanghai 200032, China

## Abstract

Adult hippocampal neurogenesis is crucial for preserving normal brain function, but how it is regulated by niche cells is uncertain. Here we show that β-arrestin 1 (β-arr1) in dentate gyrus (DG) regulates neural precursor proliferation. β-arr1 knockout (KO) mice show reduced neural precursor proliferation in subgranular zone (SGZ) which could be rescued by selective viral expression of β-arr1 but not its nuclear-function-deficient mutants under control of hGFAP promotor in DG. Compared with wild type astrocytes, β-arr1 KO astrocytes nurture less neurospheres, and this may be attributed to changed activity of soluble, heat-sensitive excretive factors, such as BMP2. RNA-sequencing reveals that β-arr1 KO DG astrocytes exhibit an aberrant gene expression profile of niche factors, including elevated transcription of *Bmp2*. Taken together, our data suggest that β-arr1 mediated nuclear signaling regulates the production of excretive factors derived from niche astrocytes and expansion of neural precursors in DG, thus maintaining homeostasis of adult hippocampal neurogenesis.

New born neurons are generated throughout whole life span in the subgranular zone (SGZ) in the dentate gyrus (DG) of adult hippocampus^1^. Emerging evidence suggests that adult hippocampal neurogenesis is involved in learning and memory, and associated with emotion regulation^2^,^3^,^4^. The adult hippocampal neurogenesis, involving multiple key processes including proliferation and differentiation of neural precursors, and maturation, migration, and functional integration of newborn neurons into the existing neural network, is believed to be regulated by a specific neuro-microenvironment called neurogenic niche in SGZ^5^.

Several niche determinants, for example, neurotransmitters such as glutamate, GABA and dopamine, non-neurotransmitters such as bone morphogenetic proteins (BMPs), sonic hedgehog (Shh), Notch, Wnt, Wnt inhibitor, growth factors, and neurotrophic factors have been found involved in adult neural precursors-niche interaction^6^. Recent work revealed that niche cells could regulate the properties of neural precursors through secretion of certain niche factors (environmental cues). For example, Song *et al.* reported that parvalbumin-expressing interneurons could dictate neural stem cells choice between quiescence and activation by secreting GABA in SGZ^7^. As a well-known type of niche cells, astrocytes are also able to sustain neural precursors and believed to be a predominant source of many adhesive and soluble niche factors^8^. Studies have shown the roles of astrocytes in hippocampal neurogenic niche^9^,^10^,^11^. Transcriptional regulation of expression levels of neural factors could be a critical mechanism to ensure the formation of the right niche, in response to environmental changes, whereas the key signaling molecules or pathways regulate niche factors required for the proliferation of neural precursor cells in adult brain are largely unknown.

β-arrestins (β-arrs), consisting of β-arr1 and β-arr2, are key regulators and mediators of G-protein coupled signaling processes. Both β-arr subtypes are expressed in many brain areas, including hippocampus^12^. Previous studies have established that β-arrs attenuate GPCR mediated signal transduction leading to receptor internalization in response to agonist stimulation^13^,^14^. β-arrs have also been shown to serve as scaffold proteins that interact with many different signaling molecules in GPCR or non GPCR signaling^15^,^16^,^17^. Our previous research demonstrated that distinct from β-arr2, β-arr1 possesses a nuclear localization sequence and could translocate into the nucleus acting as a transcriptional regulator via interaction with transcription factors, such as p300 and PPARγ^18^,^19^,^20^. In the current study, we demonstrate that β-arr1 in DG participates in adult hippocampal neurogenesis. We further propose that β-arr1-mediated signaling in niche astrocytes enhances the mitotic expansion of neural precursors in adult hippocampus and this may involve nuclear β-arr1-mediated transcription of excretive niche factors, such as BMP2, from astrocytes in neurogenic niche.

## Results

### The Ablation of β-arr1 Results in a Decrease in Proliferating Cells in Adult SGZ

**Figure 1:**
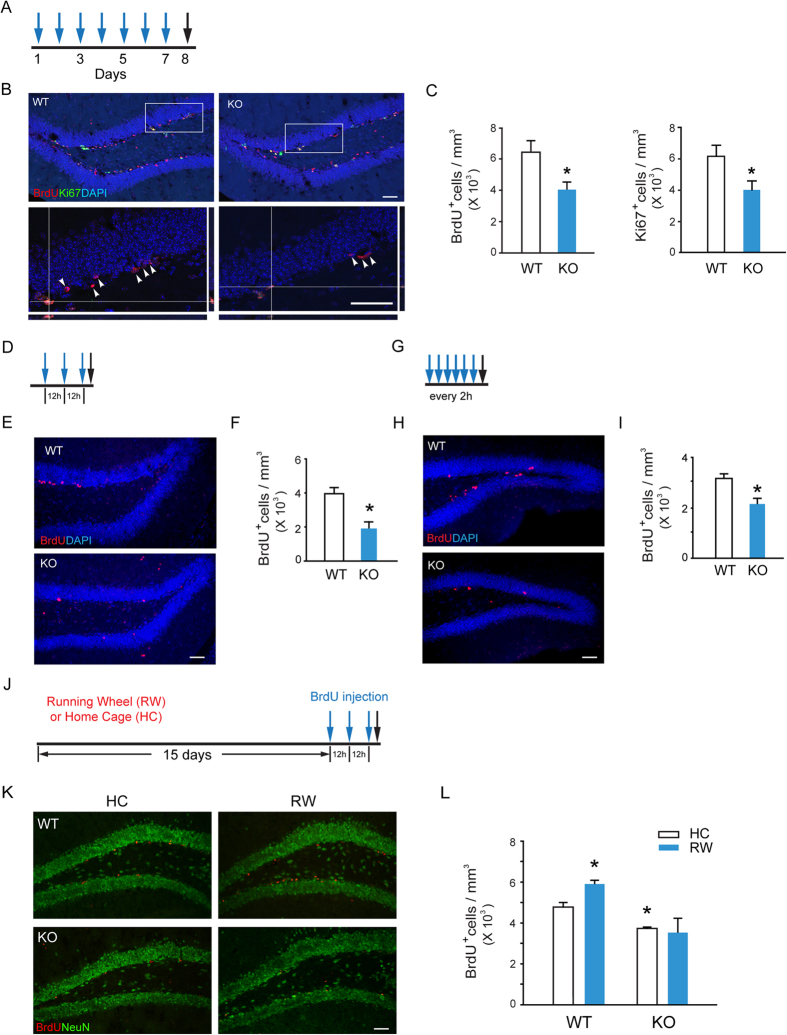
Decreased Proliferation of Neural Precursors in the SGZ of Adult β-arr1 KO mice. β-arr1 KO mice (KO) and wild type littermates (WT) of 2–3 month old were used. (**A**–**I**) Mice were injected intraperitoneally with 100 mg/kg BrdU (blue arrows) and sacrificed (black arrows) at time points indicated (**A**,**D**,**G**). Sample projected confocal images were shown (**B**,**E**,**H**) and stereological quantification (**C**,**F**,**I**) of BrdU-positive and Ki67-positive (**C**) cells in SGZ of these mice were performed. Arrowheads indicate the localization of BrdU^+^ cells. Orthogonal views are samples of BrdU^+^ Ki67^+^ cells. The cell number was normalized to the GCL (granular cell layer) volume (in mm^3^). Data represent mean ± s.e.m.; n = 3 or 4 mice for each genotype; *t*- test, *p < 0.05. Scale bar, 50 μm; (**J**–**L**) After running wheel training (RW) or housed in home cage (HC) for 15 days, mice were injected with BrdU for 3 times (blue arrows) with an 12 h interval and sacrificed (black arrow) 2 h after the last BrdU injection (**J**). Immunostaining (**K**) and stereological quantification (**L**) of BrdU^+^ cells in SGZ were performed. The cell number was normalized to the GCL volume (in mm^3^). Data represent mean ± s.e.m.; n = 3 mice/group; *p < 0.05 vs. WT/HC, two-way ANOVA, post hoc Tukey test; Scale bar, 50 μm.

The cell expansion in SGZ is critical for the maintenance of neural precursor cell pool. To explore the potential role of β-arr1-mediated signaling in hippocampal neurogenesis, the wild type (WT) and β-arr1 knockout (KO) mice were injected with 5-bromo-2′-deoxyuridine (BrdU,100 mg/kg, i.p.) once a day for 7 days and sacrificed 24 h after the last injection (Fig. 1A). We found that the number of BrdU-positive cells was decreased by ~30% in SGZ of β-arr1 KO mice [Fig. 1B,C left, t (6) = 2.755, *p* = 0.033 vs. WT], but not changed in β-arr2 KO mice (Fig. S1A–C). BrdU-positive cell number in heterozygous littermates was also determined (Fig. S1D,E). The cells that stain positive for Ki67, a marker of proliferating cells, were also decreased after the ablation of β-arr1 [t (12) = 2.361, *p* = 0.036] (Fig. 1B,C right). Two acute BrdU injection protocols were also used (Fig. 1D,G). SGZ samples taken 2 h after 3 BrdU injections with an 12 h interval showed that the BrdU-positive cell number in β-arr1 KO mice was significantly lower than that of WT mice [t (4) = 3.847, *p* = 0.018] (Fig. 1D–F). Similar result was also obtained after the injection of BrdU for 6 times in 2 h interval [t (4) = 3.483, *p* = 0.025] (Fig. 1G–I). Moreover, we found that the running wheel training failed to enhance the cell proliferation and neurogenesis in SGZ of β-arr1 KO mice, in contrast to WT mice [F_genotype × treatment_ (1,15) = 5.225, *p* = 0.041] (Fig. 1J–L). BrdU incorporation assay and Ki67 immunostaining indicated that the cell proliferation in SVZ was not affected by β-arr1 ablation (Fig. S1F–H). These results suggest that ablation of β-arr1 impairs both intrinsic and stimulated capacity of cell proliferation in adult SGZ.

### β-Arr1 is Expressed in Neural Precursors and Niche Cells in Adult SGZ

**Figure 2:**
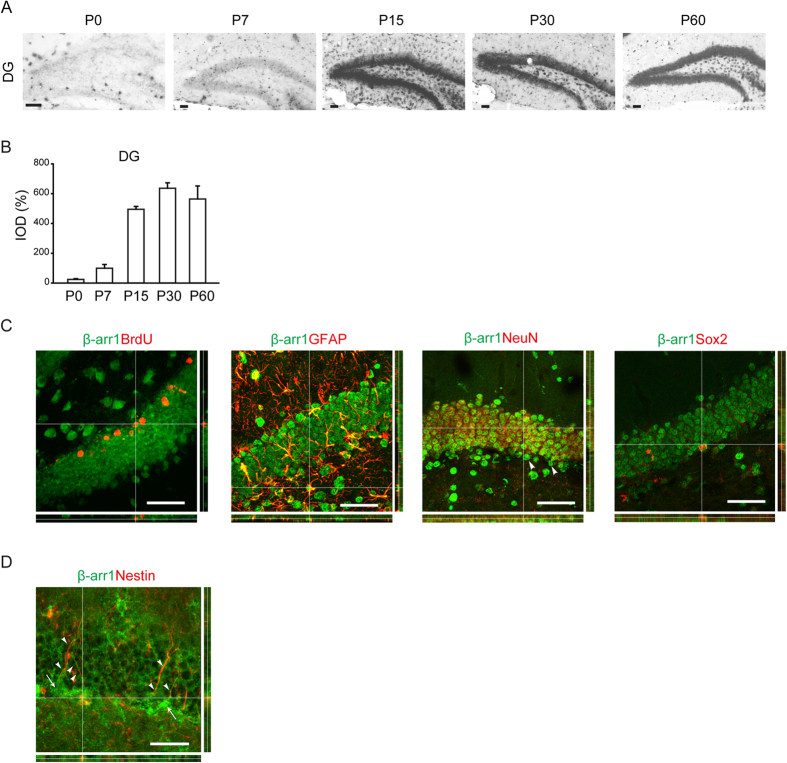
Expression of β-arr1 in Neural Precursors and Niche Cells of SGZ. (**A**,**B**) Representative images (**A**) and data of optical densities (OD) (**B**) of *in situ* hybridization using β-arr1 antisense probe on brain sections from P0, P7, P15, P30, or P60 WT mice. Scale bar, 50 μm. (**C**) *In situ* hybridization with β-arr1 antisense probe combined with immunostaining for BrdU, GFAP, NeuN, and Sox2 in the DG on brain sections of 3-month-old WT mice. Orthogonal views are shown to confirm colocalization. Arrowheads indicate β-arr1^+^ NeuN^−^ cells. (**D**) Co-immunostaining for β-arr1 and Nestin. Orthogonal views are shown to confirm colocalization. Arrows indicate the cell bodies of Nestin^+^ RGLs and arrowheads indicate their branches. Scale bar, 50 μm.

We used dioxygenin-labeled ribo-probes to examine β-arr1 mRNA levels in the hippocampus of WT mice at different postnatal age by *in situ* hybridization. We found that β-arr1 expression was low in DG at birth, but had a marked increase after postnatal day 7 (P7) and reached a plateau at P30 (Fig. 2A,B). β-arr1 expression was increased during the early-postnatal period and retained at high level in the mature DG indicating its possible role in DG development and adult function.

The result of *in situ* hybridization for β-arr1 transcript combined with immunofluorescence staining for BrdU revealed the expression of β-arr1 in the proliferating cells in SGZ (Fig. 2C). To determine whether β-arr1 is expressed in neurogenic niche, we stained brain sections with dioxygenin-labeled ribo-probes against β-arr1 and antibody against neuron marker NeuN or astrocyte marker GFAP (Fig. 2C). We observed that β-arr1 was widely expressed in the neurogenic niche cells and also a subset of NeuN-negative cells in the SGZ. Previous reports have shown that the Nestin-positive radial glial cells (RGLs) that possess the long processes extending from the SGZ towards and ramifying at the molecular layer are neural stem cells (NSCs)^21^. As shown in Fig. 2D by immunofluorescence co-staining, β-arr1 was expressed in the long processes of RGLs and co-localized with Nestin in these cells. Consistently, we also detected β-arr1 signal in Sox2, a nuclear marker of NSCs^22^ positive cells (Fig. 2C) and in WT neurospheres by Western (Fig. S2C). Altogether, these data suggest that β-arr1 is widely distributed in DG and may play a role in adult hippocampal neurogenesis.

### β-Arr1 in Adult DG Modulates the Proliferation of Neural Precursors

**Figure 3:**
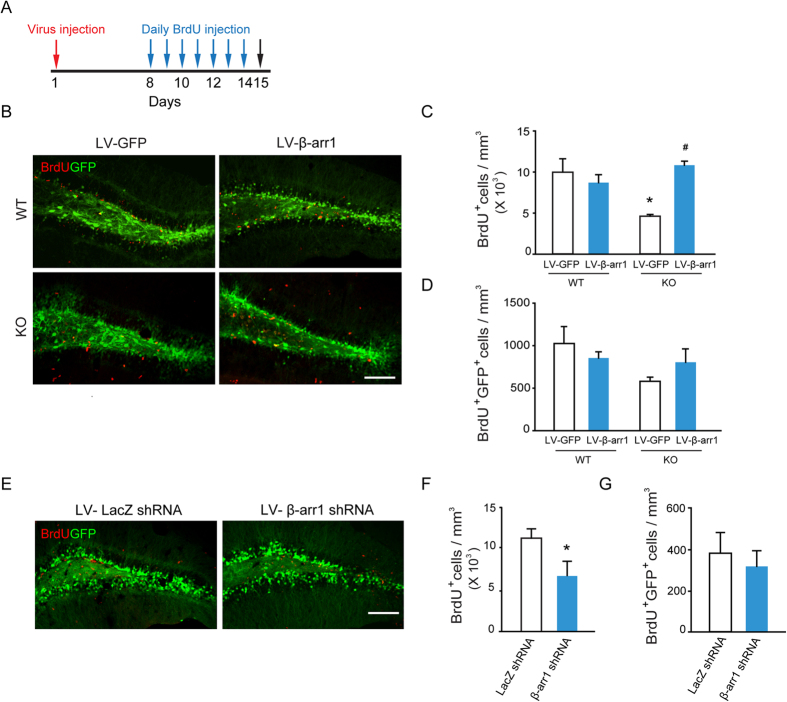
β-arr1 in the DG Regulates the Proliferation of Neural Precursors. One week after injection of lentivirus encoding GFP (LV-GFP), β-arr1 and GFP (LV-β-arr1), or GFP plus β-arr1 shRNA (LV-β-arr1 shRNA) or β-galactosidase shRNA (LV-LacZ shRNA) into the DG, mice were treated daily with BrdU (100 mg/kg i.p.) for 7 days and sacrificed 1 day after the last BrdU injection. (**A**) Schematic of experimental schedule. (**B**–**D**) Expression of LV-β-arr1 and LV-GFP. *p < 0.05 vs. WT/LV-GFP; #*p* < 0.05 vs. KO/LV-GFP, two-way ANOVA, post hoc Tukey test. (**E**–**G**) Expression of LV-β-arr1 shRNA and LV-LacZ shRNA. *p < 0.05, *t*-test, BrdU^+^ cell and BrdU^+^ GFP^+^ cell number in the SGZ are normalized to the GCL volume (in mm^3^). Values represent mean ± s.e.m.; n = 3–4 for each group; Scale bar, 100 μm.

To determine whether the impairment of cell proliferation in SGZ we observed in adult β-arr1 KO mice is due to a neurodevelopmental disruption or a specific effect mediated by β-arr1 expressed in the adult DG, we expressed β-arr1 using a lentivirus expression system in the DG of WT and β-arr1 KO mice. One week after viral injection, the β-arr1 KO mice and their WT littermates were injected with BrdU daily at the dose of 100 mg/kg for 7 days and sampled one day after the last BrdU injection (Fig. 3A). Quantitative analysis of the number of BrdU-positive cells indicated that local expression of β-arr1 had no significant effect on cell proliferation in the SGZ of adult WT mice, however, it rescued neurogenesis phenotype in the SGZ of adult β-arr1 KO mice [F_genotype × treatment_ (1,13) = 11.435, *p* = 0.007] (Fig. 3B,C). As shown in Fig. 3C, the number of BrdU-positive cells was increased in DG infected by β-arr1-expressing lentivirus to a level comparable to those of WT [*p* = 0.204, *post hoc* Tukey test]. No significant change in BrdU^+^ GFP^+^ cell number in SGZ was found among the 4 groups (Fig. 3D). Moreover, the effect of down regulating β-arr1 expression in adult DG was examined. We constructed the lentiviruses that express β-arr1 shRNA and verified its knockdown efficacy in N2A cells. As shown in Fig. S2E, β-arr1mRNA level was down-regulated by 50% [t (7) = 4.715, *p* = 0.002]. β-arr1 shRNA lentivirus (1 × 10^9^ TU/ml, 1 μl) was microinjected into the DG of WT mice, followed by BrdU injections (Fig. 3A). We quantified the number of BrdU-positive cells and found the number of proliferating cells was significantly decreased after knock-down of β-arr1 in DG [t (4) = 2.940, *p* = 0.042] (Fig. 3E,F), while the numbers of BrdU^+^ GFP^+^ cells between the two groups had no difference (Fig. 3G). These results indicate that β-arr1 in the adult DG is involved in the regulation of cell proliferation in SGZ.

### β-Arr1 Regulates NSC Activation and Neuronal Production in Adult DG

**Figure 4:**
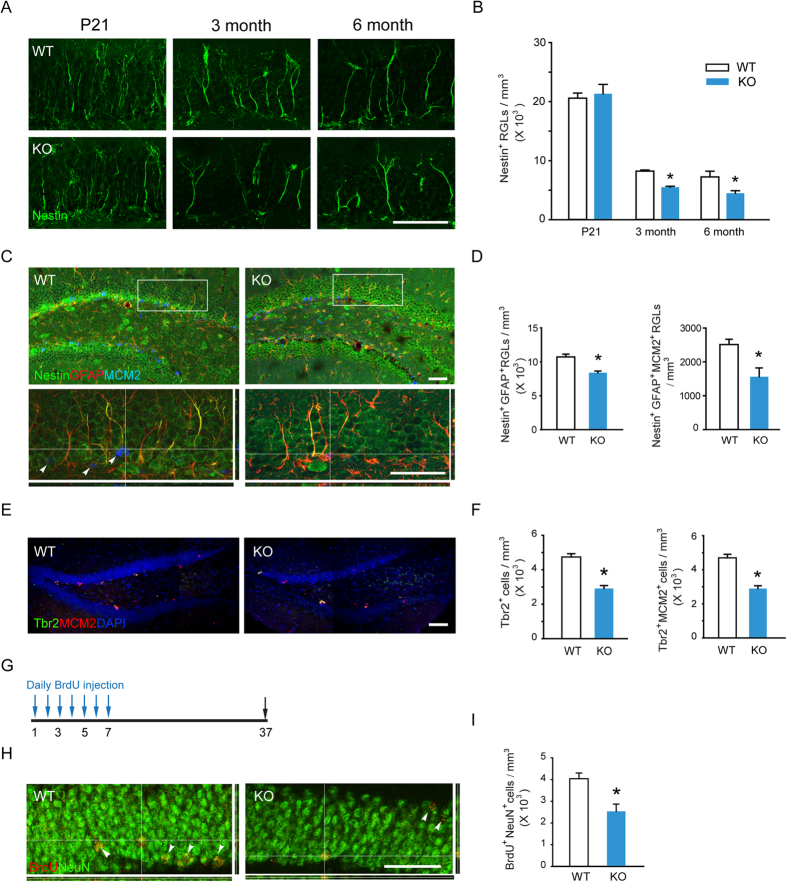
Ablation of β-arr1 Reduces NSC Activation and Neuronal Production in Adult DG. (**A**,**B**) Sample projected confocal images and stereological quantification of Nestin^+^ RGLs in DG of P21, 3 and 6 month-old mice. The number of RGLs was normalized to the GCL volume; two-way ANOVA, post hoc Tukey test. (**C**,**D**) Sample projected confocal images and stereological quantification of Nestin^+^ GFAP^+^ RGLs and Nestin^+^ GFAP^+^ MCM2^+^ RGLs in the DG of WT and KO mice. Orthogonal images ((**C**), bottom) are shown to confirm colocalization. (**E**,**F**) Confocal images and quantification of Tbr2^+^ IPCs and Tbr2^+^ MCM2^+^ IPCs in SGZ of WT and KO mice. (**G**) Schematic of experimental schedule. WT and β-arr1 KO mice were injected with BrdU (i.p., 100 mg/kg) for 7 days, and sacrificed 30 days after the last BrdU injection. (**H**,**I**) Sample projected confocal images and stereological quantification of BrdU^+^ NeuN^+^ cells in DG. High magnification orthogonal images show the colocalization of BrdU and NeuN. Data are mean ± s.e.m.; n = 5–6 per genotype; *p < 0.05 versus WT, *t*-test; Scale bar, 50 μm.

The decline of BrdU-positive cells in β-arr1 KO DG may be caused by abnormality at different neurogenesis stages. Recent study by Bonaguidi *et al.* revealed that the Nestin^+^ RGLs in SGZ are the NSCs^21^. We analyzed the number of Nestin-positive RGLs in the SGZ of both genotypes in different ages, and found that compared with those from their WT littermates, the number of Nestin-positive RGLs in SGZ was decreased in β-arr1 KO mice at the ages of 3 and 6 months [F_genotype_ (1, 24) = 6.537, *p* = 0.020] but not at P21 (Fig. 4A,B). The activation and self-renewal of NSCs are required for the maintenance of the NSC pool. We observed that Nestin^+^ GFAP^+^ RGLs were decreased in the DG of KO mice [t (4) = 5.627, *p* = 0.004] and the number of Nestin^+^ GFAP^+^ MCM2^+^ RGLs was also decreased [t (4) = 3.178, *p* = 0.003] (Fig. 4C,D). Furthermore, Nestin and BrdU double-positive RGLs were significantly fewer than those in WT mice [t (4) = 6.485, *p* = 0.003] (Fig. S4A,B). Taken together, these data indicated that β-arr1 ablation led to decreased total and activated NSCs at the population level. We found that Tbr2^+^ and Tbr2^+^ MCM2^+^ intermediate progenitor cells (IPCs) were also decreased in KO SGZ [t (4) = 7.122, *p* = 0.002 for Tbr2^+^; t (4) = 7.257, *p* = 0.002 for Tbr2^+^ MCM2^+^] (Fig. 4E,F). In order to assess the influence of β-arr1 ablation on final neuronal production in hippocampus, the number of mature newborn neurons in DG was examined^23^. Both genotypes were injected with BrdU (100 mg/kg, i.p.) once a day for 7 days and sampled 30 days after the last BrdU injection (Fig. 4G). The BrdU-positive cells double labeled with NeuN and located in DG were counted as mature newborn neurons. We found that the number of these cells was significantly decreased in KO mice compared with WT mice [t (9) = 3.540, *p* = 0.006] (Fig. 4H,I), indicating a deficiency in neurogenesis.

### β-Arr1-mediated Nuclear Signaling in DG Regulates the Proliferation of Adult Neural Precursors

The behavior of adult neural stem cells and more restricted progenitor cells in SGZ niche is modulated by both intrinsic and extrinsic factors. β-arr1 may be critically involved in adult hippocampal homeostasis via both cell-autonomous regulation and niche factor-mediated non cell-autonomous mechanism. We used *in vitro* neurosphere assay to examine the role of β-arr1 in cell-autonomous mechanism. The hippocampi from P1 mice and DG from 2-month- and 1-year-old mice were dissected to culture neurospheres. After cultured for 7 days, the size and number of neurospheres derived from both genotypes were measured and compared. There was no difference in the diameter of neurospheres derived from 2-month-old β-arr1 KO and WT DG (Fig. S4A,B), while the neurospheres derived from β-arr1 KO DG were more than those from WT littermates [Fig. S4C,D, t (6) = −3.719, *p* = 0.010].

**Figure 5:**
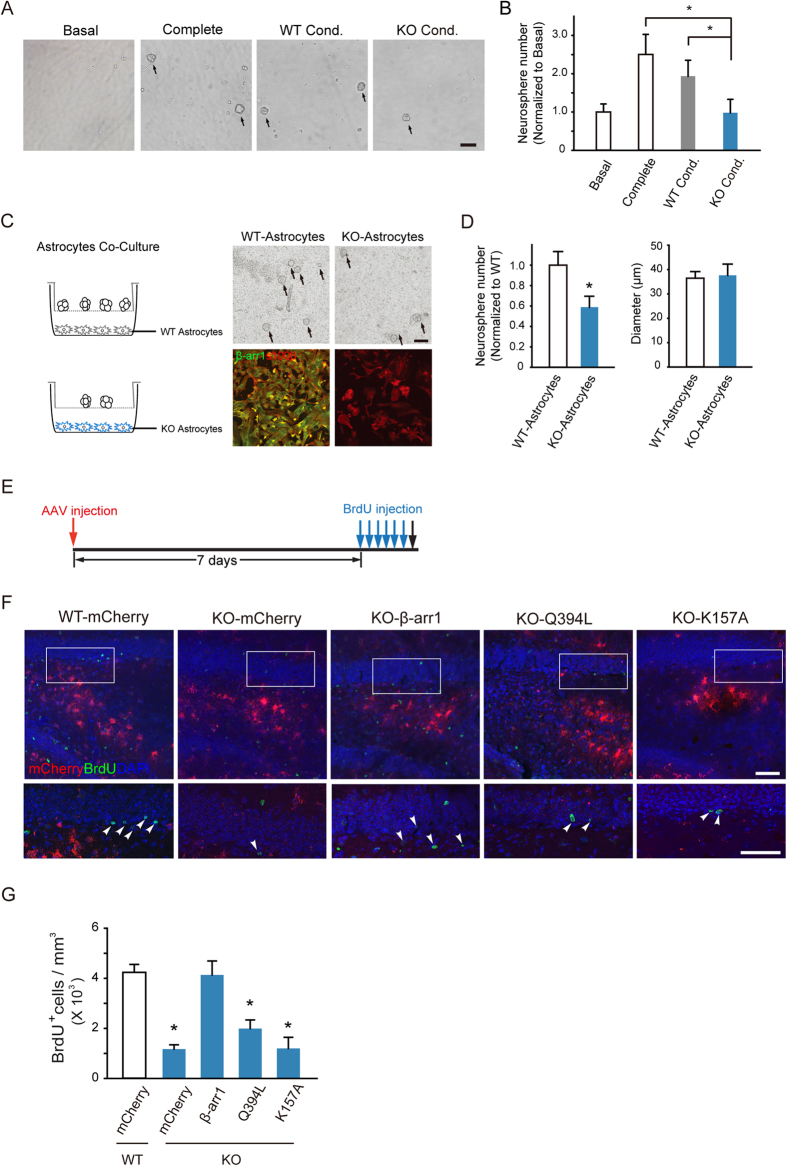
Nuclear β-arr1 Regulates the Proliferation of Adult Neural Precursors in DG. (**A**,**B**) Tissue-conditioned media derived from DG of β-arr1 KO mice sustained less WT neurospheres than the complete media and the tissue-conditioned media derived from DG of WT mice. Data are normalized to basal value, n = 3 independent experiments. One-way ANOVA; Scale bar, 100 μm. (**C**,**D**) WT neurospheres co-cultured with β-arr1 KO DG astrocytes (KO-Astrocytes) were less than those co-cultured with WT (WT-Astrocytes). Data are normalized to WT, all error bars show s.e.m. of triplicated cultures (3–4 samples per group), *t*-test; Scale bar, 100 μm. (**E**–**G**) The schematic of experimental schedule (**E**), sample projected confocal images (**F**), and stereological quantification (**G**) of BrdU^+^ proliferating cells (arrowheads) in the SGZ of WT mice injected with AAV-hGFAP-mCherry (WT-mCherry) or β-arr1 KO mice injected with AAV-hGFAP-mCherry (KO-mCherry), AAV-hGFAP-β-arr1-mCherry (KO-β-arr1), AAV-hGFAP-β-arr1Q394L-mCherry (KO-Q394L) or AAV-hGFAP-β-arr1K157A-mCherry (KO-K157A). n = 3–6 for each group; Data represent mean ± s.e.m.; *p < 0.05 *vs.* WT-mCherry; one-way ANOVA, post hoc Tukey test; Scale bar, 50 μm.

To assess the possible role of β-arr1 in maintaining the *in vivo* SGZ neural stem cell niche, we carried out tissue-conditioned media culture assay^24^. The cells dissociated from WT primary neurospheres were incubated in the tissue-conditioned media derived from β-arr1 KO or WT DG and the formed secondary neurospheres were quantified. As shown in Fig. 5A,B, the culture with WT DG-conditioned media (WT DG Cond) yielded comparable amount of neurospheres to that of complete medium, but the conditioned media derived from β-arr1 KO DG (KO DG Cond) did not [F_group_ (3, 14) = 13.853, *p* < 0.001]. Additionally, as shown in Fig. S5, neither the heat-treated WT DG-conditioned media (WT DG Heat) nor conditioned media derived from non-neurogenic WT cortex (WT Ctx Cond) could support the growth of neurospheres. These results indicate that a soluble, heat-sensitive component(s) required for neural precursor cell proliferation might be deficient in β-arr1 KO DG.

The soluble neural factors in stem cell niche are secreted by the niche cells and delivered by blood vessel. The astrocyte is one of the most important niche cells^9^. We co-incubated the wild type primary neurospheres with astrocytes derived from the DG (niche astrocytes) of WT or β-arr1 KO mice. As shown in Fig. 5C,D, the number of neurospheres produced in co-culture with β-arr1 deficient niche astrocytes was reduced by ~50% compared to those with WT niche astrocytes [t (10) = 2.405, *p* = 0.037] (Fig. 5C,D).

Our earlier study demonstrated that a distinct function of β-arr1 is translocation from the cytoplasm to the nucleus, where it acts as a transcription cofactor, to regulate gene expression^18^. Recombinant adeno-associated viruses encoding WT β-arr1, Q394L and K157A, two β-arr1 mutants incapable to translocate to the nucleus and thus deficient in transcriptional function^18^,^19^ were constructed under the control of a human GFAP promoter. One week after DG viral infection, WT or β-arr1 KO mice were injected with BrdU (6 times, once every 2 h, Fig. 5E). Injection of AAV-hGFAP-β-arr1-mCherry (KO-β-arr1) into DG of KO mice rescued the neural precursor proliferation, while expression of either Q394L or K157A failed to do so [F_group_ (4, 18) = 20.376, *p* < 0.001; *post-hoc* Tukey: *p* = 0.003 for Q394L, *p* < 0.001 for K157A *vs.* WT] (Fig. 5F,G; S6A). These data suggest that β-arr1 might regulate the neural precursor proliferation through, at least in part, its function in niche astrocytes, and β-arr1-mediated gene transcription, but not cytoplasmic or membrane function was crucial.

### RNA-Sequencing Transcriptome Shows Abnormal Gene Profiles in β-arr1 KO Niche Astrocytes

Primary niche astrocytes cultured from DG of WT and β-arr1 KO mice were subjected to transcriptional profiling by RNA-sequencing (RNA-seq) to reveal differences in gene expression. Out of 12761 genes analyzed, changed expression was detected in 1286 genes [fold change >3/2 (1.5) or <2/3 (0.6667), β-arr1 KO vs. WT] (See Supplemental Tables 2 and 3). A reduction in β-arr1 (*Arrb1*) transcript level was observed, and house-keeping genes, such as *Actb* and astrocyte marker *Gfap,* remained unchanged (Fig. S7A).

**Figure 6:**
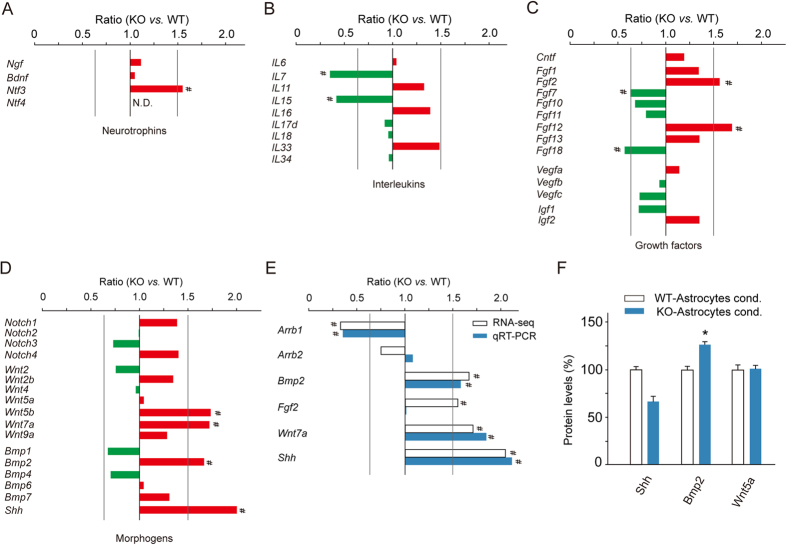
RNA-Sequencing Transcriptome Shows Abnormal Gene Profiles in β-arr1 KO Niche Astrocytes. (**A**–**D**) Changes of mRNA levels of different genes in WT and KO niche astrocytes determined by RNA-seq; (**E**) Comparison of mRNA levels of neural factors in WT and KO niche astrocytes determined by RNA-seq and qRT-PCR; Samples were collected from three mice in each group; # indicates ratio of β-arr1 KO *vs.* WT >3/2 or <2/3. (**F**) Protein levels of SHH, BMP2, and WNT5a in KO and WT astrocyte-conditioned media determined by ELISA. n = 3 independent experiments. Data were normalized to WT and represented mean ± s.e.m.; *p < 0.05 vs. WT; *t*-test.

Recent studies have found that secreted and adhesive factors are critical for neural precursor behavior. Changes of mRNA levels of several well-established niche factors, including neurotropic factors, interleukins, growth factors, and morphogens, in β-arr1 KO astrocytes were shown in Fig. 6A–D. Among neurotropic factors, the mRNA level of BDNF, a well-known neurogenic factor was not changed (Fig. 6A). A number of interleukins serve as niche regulators of adult hippocampal neurogenesis^25^. Among these 9 interleukins detected by RNA-seq, we found that mRNA levels of *Il7* and *Il15* were decreased in β-arr1 KO astrocytes. *Il15* was shown to be a neurogenic factor recently^26^. Morphogens, such as Notch, Wnt, Bmp families, and Shh, are also important members of niche factors. Changes in levels of *Wnt5b*, *Wnt7a*, *Bmp2*, and *Shh* in β-arr1 KO niche astrocytes were detected. Among them, *Bmp2*, *Shh* and *Wnt7a* were reported previously to be related to neural precursor cell expansion in DG^27^,^28^,^29^ and their changes were confirmed by qRT-PCR (Fig. 6E). ELISA data revealed elevated BMP2 [t (4) = −19.273, p < 0.001] and decreasing trend of SHH in β-arr1 KO niche astrocyte-conditioned media (Fig. 6F). The increased protein level of BMP2 in β-arr1 KO astrocyte-conditioned media conforms to the elevated level of *Bmp2* transcript in β-arr1 KO niche astrocytes detected by RNA-seq and qRT-PCR, suggesting that its expression might be under stringent transcriptional control of β-arr1. Considering the anti-mitotic effect of BMP2, the reduction of neural precursor proliferation would be a reasonable consequence of increased level of BMP2 in β-arr1 KO DG or β-arr1 KO astrocyte-conditioned media. As to *Shh* and *Wnt7a*, combining ELISA and *in vivo* data, their elevations might be the result of compensational enhancement of transcription.

Additionally, genes regulated by β-arr1 as indicated by RNA-seq were further analyzed using ChEA tool integrated in Enrichr^30^ to predict possible transcriptional regulatory machineries involved. Prediction indicated that the transcriptional activities of several transcription factors were elevated in β-arr1 KO niche astrocytes, including PPARγ (overlap: 57/3562, p < 0.01) (Supplemental Table 4). It was reported previously that β-arr1 played an inhibitory role in PPARγ signaling^31^. Those down-regulated genes in β-arr1 KO niche astrocytes might be regulated by transcription factors such as Suz12 (overlap: 125/4353, p < 0.001) as reported before^32^. Bioinformatic analysis also showed that several PPARγ-specific motifs were presented in the promoter regions of *Bmp2* (Fig. S7B). PPARγ was reported to interact directly with nuclear β-arr1 to suppress its transcriptional activity^20^. We postulate that BMP2 secreted from niche astrocytes might be under β-arr1-mediated transcriptional regulation.

## Discussion

The dynamic interaction between neural precursors and their niche is critical for neural precursors and their progeny in sensing and responding to the changes of external environment during adulthood^33^. The underlying mechanisms during this interaction remain unclear. In this study, we showed that the proliferation of neural precursors in adult SGZ under basal condition and after external stimulation was both decreased after ablation of β-arr1, an important co-factor of transcriptional regulation. Expression of the wild type β-arr1 under the control of human GFAP promotor could enhance the cell proliferation in SGZ of β-arr1 KO mice, while nuclear-function-loss Q394L and K157A mutants of β-arr1 could not. Moreover, neurosphere co-culture assays indicated that compared with the wild type astrocytes, β-arr1 KO astrocytes nurtured less neurospheres, and this might be attributed to the abnormality in soluble, heat-sensitive factors secreted from astrocytes, such as BMP2. Astrocytes are important sources of niche factors and play a key role in regulating adult neurogenesis *in vivo* via juxtacrine and paracrine signaling^8^,^9^. We propose a possible mechanism by which β-arr1 signaling in DG induces adaptation and supports the homeostasis of neural precursor cells. In this model, β-arr1 acts as a niche modulating molecule bridging the extrinsic signals and inner neural adaptive changes in adult hippocampal neurogenesis.

Neurogenesis occurs throughout the early development stage and also after maturation. Recent studies have revealed that several signaling pathways important for development, such as TGFβ superfamily, Wnt, Shh and Notch are required for neurogenesis during adulthood^6^. Mechanistic details are needed to reveal the dynamic of these signaling pathways in neurogenesis during adulthood. β-arrs have been implicated as important signaling mediators of Wnt, Shh, and Notch pathways^34^. In this study, our *in vivo* BrdU incorporation data showed that 2-month-old β-arr1 KO mice exhibited reduced cell proliferation in SGZ but not SVZ. After ablation of β-arr1, the proliferation of neural precursors in SGZ under both basal condition and after external stimulation was decreased. By *in situ* hybridization, we detected potent and wide expression of β-arr1 in mature DG and an increasing trend during postnatal development which is similar with other reports^12^,^35^. Consistently, Nestin^+^ RGLs in DG of P21 β-arr1 KO mice showed no obvious defect. These data support the notion that β-arr1 may be specifically involved in the hippocampal neurogenesis during adulthood. The adult hippocampal neurogenesis is comprised of several stages which recapitulate the complete process of neuronal development in embryonic period. Among them, the self-renewal of NSCs (RGLs) is critical for the sustainable neurogenesis in SGZ^33^. Using Nestin/GFAP/MCM2 co-staining, we found that the ablation of β-arr1 resulted in a decrease of the activated NSCs and total number of Nestin^+^ GFAP^+^ RGLs in adult animals. The Tbr2^+^ and Tbr2^+^ MCM2^+^ IPCs were also decreased. These data indicate decreased stem cell activation and expansion of progenitors in DG after β-arr1 systemic knockout, which may be interpreted as increased cell cycle exit. Furthermore we found that final neuronal production in hippocampi of KO mice was decreased.

To assess whether β-arr1 mediated regulatory mechanisms are intrinsic or extrinsic, we tested β-arr1 co-localization with different cell type markers and found that β-arr1 was widely distributed in many cell types in DG including both NSCs and niche cells. In Fig. 3, after re-expression of β-arr1 in KO DG, BrdU^+^ GFP^+^ cells had a slight increase, and in lentivirus knockdown assay BrdU^+^ GFP^+^ cells also showed decreasing trend. The effects we observed might be attributed to β-arr1 in both proliferating cells and niche cells. Meanwhile, we used an AAV gene delivering system which reported before^36^,^37^ could deliver genes into astrocytes under control of a synthetic promoter derived from the human GFAP^38^ in order to re-express β-arr1 in KO niche astrocytes (KO-β-arr1 group) and the rescue effect was shown in Fig. 5E–G. Additionally, the neurospheres co-cultured with β-arr1 KO DG astrocytes were decreased, consistent with the phenomena we observed *in vivo*, however the neurospheres cultured from β-arr1 KO DG were increased in *in vitro* assays (Fig. S4). Taken together, these data suggest that β-arr1 in niche astrocytes might play a role in positive modulation of neural precursor expansion, but did not exclude the possibility that β-arr1 may also function in other types of niche cells or NSCs.

RNA-seq analysis of β-arr1 KO astrocytes revealed an abnormal profile of transcript levels of several secreted factors. Among those morphogens and growth factors previously reported to regulate neural precursor proliferation in hippocampi, both mRNA and protein levels of BMP2, a well-known anti-mitotic morphogen^39^, were increased in β-arr1 KO astrocytes and the conditioned media derived from β-arr1 KO astrocytes, suggesting that it might be a possible regulatory target of β-arr1 in the astrocyte-neural precursor interaction. Our previous work has shown that unlike β-arr2, β-arr1 is able to enter the nucleus and thus possesses a unique transcriptional regulation function^18^. We observed that BrdU-positive cells was decreased in DG of β-arr1 KO mice, but not changed in β-arr2 KO mice. We also found that the deficiency of cell proliferation in β-arr1 KO mice could be rescued by DG expression of wild type β-arr1 using AAV-hGFAP-β-arr1-mCherry, but not the expression of Q394L or K157A mutant which is unable to enter the nucleus and has no transcriptional activity^18^,^19^. It is likely that β-arr1 regulates the proliferation of neural precursors through a nuclear mechanism, analogous to known intracellular transcriptional regulators of adult neurogenesis, such as p21, Sox2, and TLX^22^,^29^,^40^. Zhuang *et al.* demonstrated that β-arr1 could suppress PPARγ function via direct interaction with PPARγ in the nucleus^20^. Earlier study has shown that PPARγ could form a heterodimer with the 9-cis-retinoic acid receptor (RXR) and exert its transcriptional promoter activity^41^. In activated macrophages, PPARγ functions as a transcriptional repressor of the inflammatory genes^42^. Our bioinformatic analysis revealed two PPARγ-specific binding motifs presented in the promoter region of *Bmp2*. Combining the data above, we hypothesize that the production of BMP2 in niche astrocytes is regulated by a β-arr1-dependent transcriptional mechanism, likely through its dynamic association with PPARγ (Fig. S7B), the detailed underlying mechanisms need to be further elucidated.

## Methods

### Animals

β-arr1 and β-arr2 KO mice generated by the laboratory of R. J. Lefkowitz (Duke University Medical Center, Durham, NC) were backcrossed onto a C57BL/6J background for 10 generations. WT, β-arr1 KO, or β-arr2 KO littermates resulted from heterozygous breeding and descendants of mice that were separately bred as homozygous WT, β-arr1 KO, or β-arr2 KO mice after the initial backcrossing were used. All mice were maintained on a 12 h light/dark cycle with food and water available *ad libitum*. All experiments were strictly in accordance with the National Institutes of Health Guide for the Care and Use of Laboratory Animals and were approved by Animal Care and Use Committee of Shanghai Medical College, Fudan University.

### BrdU Incorporation Assay *in vivo*

BrdU incorporation assay was done essentially as reported^43^,^44^,^45^. Mice were injected intraperitoneally with BrdU (Sigma Aldrich) under different BrdU concentration and injection schedule according to the BrdU protocol mentioned in the context and sampled at indicated time points (described in Immunofluorescence staining procedure). Coronal brain sections were treated with 2 N HCl solution for 30 min at 37 °C, followed by rinse in boric acid buffer and washes in PBS. Sections were incubated with BrdU antibody (AbD Serotec, 1:500) overnight at 4 °C, and then with the secondary antibody (Rhodamine conjugated donkey anti-rat, Jackson ImmunoResearch, 1:500) for 1.5 h at room temperature. 4′,6-diamidino-2-phenylindole (DAPI) was used to counterstain nuclei. The sections were then transferred to the slices and mounted with mounting medium (Thermo Fisher Scientific). The images were captured by Zeiss LSM 510 laser confocal fluorescence microscope (Carl Zeiss).

### Stereology

An unbiased design-based stereology was used in cell counting to avoid oversampling^45^,^46^,^47^. We collected sections following a fractionator principle. Staining and quantification were performed on every sixth section from Bregma + 1.54 mm to −0.22 mm (SVZ) and from Bregma −1.22 mm to −3.16 mm (DG). The positive cells (such as BrdU^+^) were counted from the center region of sections in a blinded manner under Zeiss LSM 510 laser confocal fluorescence microscope with a 5 μm guard zone. In DG, the number of positive cells was normalized by the granular cell layer volume (in mm^3^) determined by Image-Pro Plus (MediaCybernetics) following Cavalieri Principle.

### Immunofluorescence staining

Immunofluorescence staining on mouse brain section was performed as previously described^48^. In brief, the animals were anesthetized by 10% chloral hydrate and perfused intracardiacally with 40 ml normal saline, followed by 4% paraformaldehyde. Then the brains were quickly removed and fixed with 4% paraformaldehyde at 4 °C for about 4 h and stored in 30% PBS-buffered sucrose solution for 36 h. Coronal sections (40 μm) were cut with a cryostat (Leica) and washed in PBS, incubated with blocking buffer (10% donkey serum in PBS containing 0.3% Triton X-100) for 2 h, and then incubated in primary antibody diluted in the blocking buffer overnight at 4 °C. Sections were subsequently washed in PBS, incubated in the secondary antibody for 2 h at room temperature, and washed with PBS three times. As the last step, the slices were mounted with anti-quenching mounting medium (Thermo Fisher Scientific) and then the coverslips were applied. The images were captured under the Zeiss LSM 510 and analyzed by the persons blind to the genotype and treatment. The primary antibodies used for immunostaining were shown in Supplemental Methods.

### *In situ* Hybridization

The *in situ* hybridization of free-floating brain sections was performed as described previously^49^. The sequences of probes were designed according to the Allen Brain Atlas probe database (ID: RP_050609_04_B01). The partial mouse cDNA fragment was obtained by reverse-transcription polymerase chain reaction (TaKaRa) and cloned into vector pcDNA3.0. After verification of the sequence, the antisense and sense digoxigenin (DIG)-labeled RNA riboprobes were synthesized using DIG RNA Labeling Kit (Roche). The 30 μm brain sections were fixed by 4% paraformaldehyde in PBS after treated with 2–5 μg/ml proteinase K for 20 min at 37 °C, pre-hybridized with hybridization buffer containing 50% formamide (v/v) deionized, 5 × SSC, 0.02% SDS (w/v), 2% blocking solution (Roche) for 1 h and then hybridized with riboprobe (50 ng per brain section) in hybridization buffer at 55 °C overnight. After high stringency washing, immunological detection of DIG was done by incubation of the brain sections with anti-DIG antibody fragment conjugated with alkaline phosphatase (AP) or fluorescein (1:200) at room temperature for 3 h. The AP sections were developed with NBT/BCIP Stock Solution (Roche) at room temperature for 2–3 h and then analyzed under a phase contrast microscope (Olympus). The fluorescein sections were subjected to Immunofluorescence co-staining or analyzed by confocal fluorescence microscope directly.

### Microinjection and Viral Infection in DG

The recombinant lentiviruses were constructed according to the described method^50^. The vectors encoding GFP-tagged full-length of β-arr1 (LV-β-arr1) and GFP-tagged β-galactosidase (LV-GFP) were kindly provided by Dr. Gang Pei (Tongji University). Construction of pBS/U6/β-arr1 shRNA and determination of knockdown efficacy were done based on our published method^18^. The titers of these lentiviruses were about 1 × 10^9^ transducing units (TU)/ml. The AAV plasmids were designed and constructed based on pAAV-hGFAP-ChR2 (H134R)-mCherry from Karl Deisseroth (Addgene plasmid # 27055) as described^18^,^38^, and packaged by Neuron Biotech (Shanghai) Co., Ltd. as AAV_2/8_ (serotype 8, titer: 1 × 10^12^ vg/ml). For the microinjection of viruses, the anesthetized mice were positioned on a stereotaxic apparatus (NARISHIGE Scientific Instrument LAB) with the injection syringe of 34 gauge tips (Hamilton Bonaduz AG) aimed at DG. The intended stereotaxic coordinates were: AP:−2.00 mm; ML: ± 1.6 mm; DV:−2.0 mm and AP:−3.00 mm; ML: ± 2.6 mm; DV:−3.2 mm. 1 μl of the lentivirus was infused into DG at a rate of 0.2 μl/min (0.5 μl at a rate of 0.1 μl/min for AAVs). The needle was left in place for additional 5 min. The BrdU incorporation assay was carried out after a 1 week recovery.

### Neurosphere Culture

Primary neurosphere was cultured as described previously^51^. In brief, the DG was dissected and finely minced, digested for 20 min at 37 °C in Neural Stem Cell Basal Medium (Stem Cell Technology) added with PDD [2.5 U/ml papain (Worthington), 1 U/ml dispase (Roche), and 250 U/ml DNase I (Sigma)] and mechanically dissociated using a Pasteur pipette with a fire-polished tip. NSCs were purified with two Percoll gradients (25% then 65%, GE) and plated at a density of 10^5^ cells/cm^2^ in NSC Basal Medium with NSC Proliferation Supplements (10×), bFGF (20 ng/ml) and EGF (20 ng/ml) (Stem Cell Technology). Cells were incubated at 37 °C in 5% CO_2_ at 95% humidity for 7 days and then the number and diameter of the neurospheres were analyzed by Image-Pro Plus (MediaCybernetics). The medium was changed every 2 days.

### Tissue-conditioned Media Culture Assay

This assay was done as described previously with minor modifications^24^. DG of 2 month-old KO and WT mice was dissected according to previous report^52^ in approximately equal mass in oxygen-saturated ACSF (119 NaCl, 2.5 KCl, 2.5 CaCl_2_, 1.0 MgSO_4_, 1.25 NaH_2_PO_4_, 26.0 NaHCO_3_, and 10 glucose in mM, pH 7.3–7.4) and incubated in 250 μl of Neural Stem Cell Basal Medium (Stem Cell Technology) without EGF or bFGF at 37 °C for 24 h. The tissue-conditioned media from two mice DG were gathered and centrifuged, the supernatant was used to incubate the dissociated primary neurospheres from WT DG in 24-well culture plate. The primary neurospheres were plated at low density (2000 cells/ml) and incubated at 37 °C in 5% CO_2_ and 20% O_2_ at 95% humidity. The medium was changed every 2 days. The number of secondary neurospheres generated was counted at DIV 7 under microscopy (Olympus).

### Primary Astrocyte Culture and Neurosphere Co-culture

Primary astrocyte cultures were generated essentially as described previously^9^,^53^. Briefly, isolated adult dentate gyrus was transferred to Earle’s Balanced Salt Solution containing PDD and incubated for 20 min at 37 °C, then rinsed in DMEM/F12 medium (1:1 v/v; Life Technologies) and triturated carefully with the fire-polished Pasteur pipette to a single-cell suspension. The isolated cells were re-suspended and seeded onto Matrigel-treated wells (Matrigel; BD) in astrocyte medium (DMEM/F12 medium containing 2 mM L-glutamine, 0.6% glucose, 5.2 ng/ml sodium selenite, 0.025 mg/ml insulin and 0.1mg/ml transferrin, supplemented with 10% FBS). These samples were cultured at 37 °C with 5% CO_2_ for 6 days (the medium was changed every 2 days) and then shaken at 100 rotations/min for 3 h at 22 °C to dissociate proliferating cells, oligodendrocyte progenitors, and neurons from bottom of the well. The purity of the astrocyte cultures was evaluated by immunostaining for s100β (Sigma, 1:200). For co-culture experiments, 2 h after the astrocyte culture medium in these wells was replaced by Neural Stem Cell Basal Medium, a transwell insert (0.4 mm pore, 12 mm diameter; Millipore) was placed and NSCs were seeded in the upper compartment and co-cultured with astrocytes in the lower compartment. 50% of medium was changed every 3 days. The number of neurospheres was calculated under a phase contrast microscope (Olympus) and images were captured for further analysis.

### Running Wheel Training

This training was done as previously described^54^. The mice in running wheel group get access to wheel for 15 days and then subjected to BrdU incorporation assay.

### Transcriptome Analysis

Astrocyte samples cultured from KO and WT DG (1 × 10^6^ cells) were collected in TRIzol (Life Technologies) and processed according to the manufacturer’s instructions. Total RNA was purified and quality checked on Agilent 2100 Bioanalyzer. The RNA 260/280 ratios were >1.8 and RIN were 9.8 and 10.0. Library construction and sequencing were performed by WuXi AppTec. Twenty million 2 × 100bp reads were acquired. 89.6% and 90.4% bases reached a Phred quality score of 30. Short reads were aligned to the mouse reference genome (mm9) using Tophat^55^. Transcript expression estimation and differential analysis were done using Cufflinks^55^. The differentially expressed genes were used for gene ontology and pathway enrichment analysis. Data were further analyzed by transcriptional machinery prediction tool Enrichr^30^. The raw data have been submitted to GEO (accession number: GSE66471). An overview of sequencing quality and depth can be found in Supplemental Table 2.

### Quantitative Real time PCR (qRT-PCR)

Samples were dissected, frozen in TRIzol, and stored at −80 °C until processing. Total RNAs were extracted and 3 μg of total RNA was reverse-transcribed using the SuperScript III First-Strand cDNA Synthesis System (Invitrogen). Control reactions without reverse transcriptase were done in parallel. qRT-PCR was performed on ROTOR-GENE RG-3000A (Corbett research) using the SYBR green Premix Ex Taq system (TaKaRa) according to the manufacturer’s instructions with specific primers shown in Supplemental Table 1.

### ELISA

The astrocyte-conditioned media were collected and concentrated with Centrifugal Filter Units (10 K, Millipore) for ELISA detection. The antibodies for indirect-ELISA were purchased from Abcam (Shh, Cat no. ab19897; BMP2, Cat no. ab14933; Wnt5a, Cat no. ab72583). ELISA was done according to the manufacturer’s instructions. Briefly, the media were incubated in the plates at 4 °C overnight (duplicated for each sample) and blocked by 5% skim milk. The primary antibody was incubated for 2 h at room temperature. After washes with PBS, the secondary antibody conjugated with HRP was added. The color reaction was developed by TMB for 30 min and stopped by 2 M H_2_SO_4_. The optical density for each well was read by microplate reader (TECAN) at 450 nm.

### Statistical Analysis

Data are represented as mean ± s.e.m. and analyzed using Sigma Stat 3.5. The statistical significant was determined by One-way or Two-way Analysis of Variance (ANOVA) for multiple comparisons followed by Tukey’s post-hoc test. The two-tailed Student’s *t*-test was used for comparisons between two groups.

## Additional Information

**How to cite this article**: Tao, Y. *et al.* Astroglial β-Arrestin1-mediated Nuclear Signaling Regulates the Expansion of Neural Precursor Cells in Adult Hippocampus. *Sci. Rep.* **5**, 15506; doi: 10.1038/srep15506 (2015).

## Supplementary Material

Supplementary Information
