# Supplementary material for: Astroglial β-Arrestin1-mediated Nuclear Signaling Regulates the Expansion of Neural Precursor Cells in Adult Hippocampus
Source: Sci Rep. 2015 Oct 26;5:15506. doi: 10.1038/srep15506 (PMC4620451; doi:10.1038/srep15506)
Supplement: Supplementary Information [file srep15506-s1.pdf]

Astroglial β-Arrestin1-mediated Nuclear Signaling Regulates the Expansion of Neural Precursor Cells in Adult Hippocampus

Yezheng Tao*, Li Ma*, Zhaohui Liao, Qiumin Le, Jialing Yu, Xing Liu, Haohong Li, Yuejun Chen, Ping Zheng, Zhengang Yang, Lan Ma#

The State Key Laboratory of Medical Neurobiology, School of Basic Medical Sciences, the Institutes of Brain Science, and the Collaborative Innovation Center for Brain Science, Fudan University, Shanghai 200032, China.

*These authors contributed equally.

#Corresponding author. E-mail: lanma@fudan.edu.cn

Supplemental Methods

Antibodies for Immunofluorescence staining

These antibodies were used: rat anti-BrdU (Accurate, OBT0030G, 1:500); rabbit anti-Ki67 (Leica, NCL-Ki-67p, 1:200); mouse anti-GFAP (Millipore, MAB360, 1:500); rabbit anti-GFAP (DAKO, Z033429, 1:1000); mouse anti-NeuN (Millipore, MAB377, 1:200); chicken anti-Nestin (Ave Lab, NES, 1:500); mouse anti-Nestin (Millipore, MAB353, 1:500); mouse anti-MCM2 (Millipore, MAB1568, 1:500); rabbit anti-Tbr2 (Abcam, ab23345, 1:200), mouse anti-s100β (Sigma, S2532, 1:200); rabbit anti-β-arr1 (Abcam, ab31868, 1:200); 4',6-diamidino-2-phenylindole (DAPI) was purchased from Sigma-Aldrich (D9564); The fluorescent secondary antibodies were used at 1:500 dilutions (donkey from Jackson ImmunoResearch).

Western Blot

Western Blot was done as previously described[1, 2]. Neurospheres protein extracts were obtained by lysing in 100 µl SDS sample buffer (0.3 mmol/L Tris-HCl, pH 6.8, 30% glycerol, 10% SDS, 6% β-mercaptoethanol, and 0.012% bromophenol blue). Cell extracts were resolved on 10% SDS polyacrylamide gels and transferred onto nitrocellulose membranes. Antibodies against β-arr1 (Abcam, ab31868, 1:1000) and β-actin (Cell Signaling Technology, 4967, 1:1000) were used. The membranes were washed in Tris-buffered saline with Tween-20 (TBST) and incubated with primary antibodies overnight followed by IRDye® 700DX or 800DX conjugated anti-rabbit IgG (Rockland Immunochemicals, Gilbertsville, PA,

1:50000) for 1 h. The membranes were then scanned in the appropriate channels (Odyssey, LI-COR Biosciences).

Immunohistochemistry with DAB

Immunohistochemistry with DAB was performed as our previous report[1]. Sampling procedure was same as immunofluorescence staining (shown in Methods). The floating brain sections were incubated in primary antibody against β-arr1 (Abcam, ab31868, 1:200) at 4 ℃ overnight after antigen retrieval. The next day, after rinsed in PBS for three times, sections were incubated for 2 h with the biotinylated anti-rabbit IgG (1: 200) and then incubated at room temperature for 40 min in avidin-biotin-complex solution (ABC Solution; Vector Laboratories) in PBS. The peroxidase reaction was visualized in 0.01 M PBS containing 0.025% diaminobenzidine tetrahydrochloride (DAB) and 0.03% H_2O_2. The images were captured by an image analysis system (Spot Advanced 4.1.2, Diagnostic Instruments) on Olympus BX41 microscope.

Legends for Supplemental Figures

S1. Number of proliferating cells was not decreased in SGZ of β-arr2 KO mice and SVZ of β-arr1 KO mice. (A-C) 2-3 month-old β-arr2 KO mice and wild type littermates (WT) were injected daily with BrdU for 7 days and sacrificed 1 day after the last BrdU injection. Immunostaining and stereological quantification of BrdU-positive and Ki67-positive cells in SGZ were performed. Data are normalized to the GCL volume; *t*-test, *$p< 0.05$ *vs* WT. (D-E) 2-3 month-old β-arr1 heterozygous (HET) mice were injected daily with BrdU for 7 days and sacrificed 1 day after the last BrdU injection [F_{group} (2, 11)= 4.442, p=0.046]. Data are normalized to the GCL volume. *$p< 0.05$ *vs.* WT, One-way *ANOVA*, Tukey's post-hoc test. (F-H) Sample projected confocal images and stereological quantification of BrdU and Ki67 positive cells in adult SVZ (in cubic millimeters). LV: lateral ventricle. Data represent mean ± s.e.m.; n=3-4 /genotype. Scale bar, 50 μm.

S2. Specificity of riboprobe and antibody used for detection of β-arr1 and efficiency of β-arr1 shRNA. (A) *In situ* hybridization using β-arr1 antisense probe obtained much stronger staining than the sense probe in DG, CA1, and CA3 of wild-type mice. (B) *In situ* hybridization using β-arr1 antisense probe detected the expression of β-arr1 in WT but not in β-arr1 KO hippocampus. Scale bar, 50 μm; (C) β-arr1 was expressed in neurospheres detected by Western Blot. (D) β-arr1 was detected in WT but not KO DG using β-arr1 antibody. Arrows indicate β-arr1$^+$ RGL like cells. (E) Knock down efficacy of β-arr1 shRNA in N2A cells.

S3. Number of proliferating radial glia cells was decreased in β-arr1 KO mice. Sample projected confocal images (A) and stereological quantification (B) of Nestin$^+$ BrdU$^+$ cells in SGZ. The number was normalized to WT. Data represent mean ± s.e.m.; n=3/genotype; *$p< 0.05$ *vs.* WT, *t*-test; Scale bar, 50 μm.

S4. β-arr1 KO NSCs from adult, but not neonates hippocampus, display an increase in

primary neurospheres formation. (A-C) Representative images (A), the diameters (B), and the numbers (C) of primary hippocampal neurospheres derived from 2-month-old β-arr1 KO mice and WT littermates at different input cell density. (D) The number of primary neurospheres from hippocampi of P1 mice and DG of 2-month- and 1-year-old mice after cultured for 7days. Data represent mean ± s.e.m. from two independent experiments with three littermates for each genotype; Two-way ANOVA and *t*-test; *$p < 0.05$. Scale bar, 100μm.

S5. The effect of heat-denaturation of tissue conditioned media on neurosphere formation. WT neurospheres were cultured in basal, complete (with bFGF and EGF), conditioned (Cond), or heat-denatured conditioned (Heat) media derived from hippocampi (Hip) or cortex (Ctx) of WT or β-arr1 KO mice. Data represent mean ± s.e.m. from three independent experiments and normalized to basal media; *$p < 0.05$ vs. Basal; #$p<0.05$ *vs.* WT-Hip cond.; Two-way ANOVA.

S6. Expression of β-arr1 in infected astrocytes and the phenotypes of infected cells. (A) Shown are the orthogonal images to confirm the expression of β-arr1 in mCherry$^+$s100β$^+$ cells in KO-β-arr1 group. (B) Orthogonal view and arrowheads indicate the colocalization of mCherry and s100β (upper panel). Orthogonal view and arrowheads indicate mCherry$^+$Nestin$^+$ cells with the star-like shape in molecular layer. Arrows show mCherry$^+$Nestin$^-$ cells (lower panel).

S7. Schematic diagram of a possible mechanism by which β-arr1 regulates adult hippocampal neurogenesis. (A) Changes of mRNA levels of β-arrs, astrocyte markers, and β-actin in WT and β-arr1 KO niche astrocytes detected by RNA-seq. # indicates ratio of β-arr1 KO *vs*. WT>3/2 or <2/3. (B) β-arr1 associates with PPARγ in the nucleus of niche astrocytes and down regulates the expression of *Bmp2* and likely other factors required for the expansion of neural precursor cells, promoting neurogenesis in DG. In the absence of β-arr1, PPARγ is free to bind to *Bmp2* promoter and is able to activate the expression of *Bmp2*, which attenuates hippocampal neurogenesis in adult animal.

Table S1. Primers for qRT-PCR

Gene	5'Primer	3'Primer
Arrb1	CCTGGATGTCTTGGGTCTG	TGATGGGTTCTCCGTGGTA
Arrb2	CAGCCAGGACCAGAGGACA	TGATAAGCCGCACAGAGTT
Bmp2	GGGACCCGCTGTCTTCTAGT	TCAACTCAAATTCGCTGAGGAC
Fgf2	GCGACCCACACGTCAAACTA	TCCCTTGATAGACACAACTCCTC
Wnt7a	GCCCACCTTTCTGAAGATCA	GGGCGTACTGGTGTGTGTTGT
Shh	AAAGCTGACCCCTTTAGCCTA	TTCGGAGTTTCTTGTGATCTTCC

Table S2. Overview of RNA-seq Quality and Depth

Catalog	WT	β-arr1 KO
#Reads (Million)	20.4	21
Yield (Mbases)	2040.5	2098.7
⩾ Q30 Bases (PF, %)	89.6	90.4
Aligned Bases (Mbases)	1843	1897.3
PCT Aligned_Bases	0.903	0.904
Coding_Bases (Mbases)	1010.1	1038
UTR Bases (Mbases)	507.3	520.5
Intronic Bases (Mbases)	141.9	147.8
Intergenic Bases (Mbases)	183.7	191.1
PCT_Coding Bases	0.548	0.547
PCT UTR Bases	0.275	0.274
PCT Intronic Bases	0.077	0.078
PCT Intergenic Bases	0.1	0.101
PCT mRNA Bases	0.823	0.821
Median_5’ - 3’ Bias	1.552	1.449

Table S3. Overview of the Result of RNA-seq

Genes	Total	β-arr1 KO vs. WT DG Astrocytes		
		Changed	Up-regulated	Down-regulated
Number	12761	1286	703	583
%	100	10.1	5.5	4.6

GEO link:
http://www.ncbi.nlm.nih.gov/geo/query/acc.cgi?token=khgjuwwgdxgzbgx&acc=GSE66471

Table S4. Transcription Factor Regulation Predicted by ChEA Analysis of Genes Altered by β-arr1 Ablation (β-arr1 KO *vs.* WT DG Astrocytes)

Transcription Factor Prediction Derived from Up-regulated Genes			Transcription Factor Prediction Derived from Down-regulated Genes		
**Term**	**Overlap**	**P-value**	**Term**	**Overlap**	**P-value**
**AR-19668381 (human)**	92/3516	9.97E-15	**AR-19668381 (human)**	59/3516	8.79585E-05
**AR-20517297 (human)**	38/2047	0.001669141	**AR-20517297 (human)**	39/2047	0.000139999
**BMI1-19503595 (mouse)**	15/661	0.008525756	**BMI1-19503595 (mouse)**	12/661	0.039947841
**CDX2-19796622 (mouse)**	10/334	0.005112808	**CEBPB-20176806 (mouse)**	28/1588	0.00366122
**CLOCK-20551151 (human)**	9/407	0.041970043	**CNOT3-19339689 (mouse)**	31/1542	0.000286764
**CRX-20693478 (mouse)**	13/668	0.039857085	**DNAJC2-21179169 (human)**	24/899	2.38614E-05
**CTCF-18555785 (mouse)**	40/1568	1.54E-06	**EED-16625203 (mouse)**	31/829	1.04579E-09
**CTNNB1-20460455 (human)**	24/988	4.04E-04	**EGR1-19032775 (human)**	8/276	0.008210745
**CUX1-19635798 (human)**	55/3040	2.82E-04	**EGR1-20690147 (human)**	84/6196	0.002593573
**DNAJC2-21179169 (human)**	19/899	0.006826367	**EOMES-21245162 (human)**	17/931	0.015716309
**E2F1-17053090 (human)**	33/1725	0.002115022	**EP300-20729851 (mouse)**	43/2082	9.7334E-06
**EED-16625203 (mouse)**	46/829	2.01E-18	**EP300-21415370 (mouse)**	20/1013	0.004119498
**EGR1-20690147 (human)**	130/6196	9.60E-14	**ERG-21242973 (human)**	7/321	0.048154201
**EOMES-20176728 (mouse)**	32/1741	0.004418207	**ESR1-17901129 (mouse)**	12/443	0.002318966
**EOMES-21245162 (human)**	22/931	9.88E-04	**ESR1-21235772 (human)**	9/228	0.000691515
**EP300-20729851 (mouse)**	44/2082	4.54E-05	**EWS-ERG-20517297 (human)**	17/1038	0.038156508
**EP300-21415370 (mouse)**	22/1013	0.002741139	**EWS-FLI1-20517297 (human)**	15/574	0.000986446
**ERG-21242973 (human)**	11/321	0.001234582	**EZH2-18974828 (mouse)**	41/1298	2.58996E-10
**ESR1-20079471 (human)**	7/216	0.012131859	**FOXA2-19822575 (human)**	40/2966	0.039793187
**ESR1-21235772 (human)**	6/228	0.045612482	**GATA1-21571218 (human)**	36/2600	0.036256797
**ESR2-21235772 (human)**	12/424	0.003518481	**GATA2-20887958 (mouse)**	39/1708	2.76507E-06
**ESRRB-18555785 (mouse)**	27/1433	0.006345166	**GATA3-20176728 (mouse)**	15/834	0.025160178
**EWS-ERG-20517297 (human)**	20/1038	0.014116249	**GLI1-17442700 (mouse)**	3/73	0.040924383
**EZH2-18974828 (mouse)**	63/1298	3.78E-22	**HNF4A-19822575 (human)**	85/6079	0.000982394
**FOXA2-19822575 (human)**	57/2966	4.48E-05	**HTT-18923047 (human)**	12/566	0.014413125
**FOXP2-21765815 (mouse)**	25/1164	0.001676942	**IRF8-21731497 (mouse)**	8/319	0.017878555
**GATA1-19941827 (mouse)**	33/1834	0.005266905	**JARID2-20064375 (mouse)**	35/1117	7.08166E-09
**GATA1-21571218 (human)**	39/2600	0.037255596	**JARID2-20075857 (mouse)**	45/1258	5.63942E-13
**GATA2-19941826 (human)**	36/2401	0.044514062	**JUN-21703547 (human)**	26/1580	0.011526375
**GATA2-20887958 (mouse)**	27/1708	0.045384345	**KLF1-20508144 (mouse)**	26/1144	0.000145598
**GATA3-20176728 (mouse)**	20/834	0.001398694	**KLF4-18358816 (mouse)**	33/1696	0.000323512
**GLI1-17442700 (mouse)**	4/73	0.010388689	**KLF4-19030024 (mouse)**	38/1502	3.43859E-07
**HNF4A-19822575 (human)**	106/6079	9.76E-07	**LMO2-20887958 (mouse)**	44/1741	3.84191E-08

**IKZF1-21737484 (human)**	5/155	0.03238995	**LYL1-20887958 (mouse)**	20/752	0.000119544
**JARID2-20064375 (mouse)**	65/1117	6.19E-27	**MEIS1-20887958 (mouse)**	27/1452	0.002119956
**JARID2-20075857 (mouse)**	66/1258	5.80E-25	**MTF2-20144788 (mouse)**	74/2979	7.1727E-13
**JUN-21703547 (human)**	32/1580	0.001021991	**MYC-18940864 (human)**	15/746	0.010427155
**KLF1-20508144 (mouse)**	21/1144	0.01942168	**MYC-19915707 (human)**	53/2974	4.70992E-05
**KLF4-18358816 (mouse)**	28/1696	0.026595951	**NACC1-18358816 (mouse)**	16/769	0.006138099
**KLF4-19030024 (mouse)**	40/1502	5.44E-07	**NANOG-16153702 (human)**	33/1685	0.000288969
**LMO2-20887958 (mouse)**	32/1741	0.004418207	**NANOG-16518401 (mouse)**	56/3481	0.000390341
**MITF-21258399 (human)**	97/5574	3.73E-06	**NANOG-18347094 (mouse)**	39/1905	3.14968E-05
**MTF2-20144788 (mouse)**	132/2979	3.12E-44	**NANOG-18358816 (mouse)**	23/1231	0.004190781
**MYC-19915707 (human)**	45/2974	0.023163813	**NANOG-18692474 (mouse)**	60/3050	6.42495E-07
**MYC-20876797 (human)**	37/1406	1.95E-06	**NANOG-18700969 (mouse)**	12/344	0.000280332
**MYCN-19997598 (human)**	7/234	0.017842975	**NFE2L2-20460467 (mouse)**	17/1055	0.04319882
**NANOG-16518401 (mouse)**	54/3481	0.008473007	**NR0B1-18358816 (mouse)**	33/1689	0.000301129
**NANOG-18347094 (mouse)**	44/1905	5.55E-06	**NR1I2-20693526 (mouse)**	16/939	0.032321421
**NANOG-18358816 (mouse)**	22/1231	0.022101221	**NRF2-20460467 (mouse)**	17/1055	0.04319882
**NANOG-18692474 (mouse)**	64/3050	8.49E-07	**PAX3-FKHR-20663909 (human)**	28/1063	6.32608E-06
**NFE2L2-20460467 (mouse)**	19/1055	0.029923713	**PHC1-16625203 (mouse)**	31/922	1.15148E-08
**NR0B1-18358816 (mouse)**	27/1689	0.040591266	**POU3F2-20337985 (human)**	35/1697	7.10464E-05
**NRF2-20460467 (mouse)**	19/1055	0.029923713	**POU5F1-16153702 (human)**	17/622	0.000282525
**PAX3-FKHR-20663909 (human)**	37/1063	2.23E-09	**POU5F1-18347094 (mouse)**	43/2107	1.29138E-05
**PHC1-16625203 (mouse)**	40/922	7.95E-13	**POU5F1-18358816 (mouse)**	17/753	0.002160102
**POU3F2-20337985 (human)**	42/1697	1.76E-06	**POU5F1-18692474 (mouse)**	78/4228	1.22342E-07
**POU5F1-16153702 (human)**	16/622	0.002093531	**POU5F1-18700969 (mouse)**	14/566	0.002351943
**POU5F1-16518401 (mouse)**	29/1539	0.004749607	**PPARD-21283829 (human)**	59/3446	5.07778E-05
**POU5F1-18347094 (mouse)**	45/2107	2.89E-05	**PPARG-20176806 (mouse)**	20/837	0.00046012
**POU5F1-18692474 (mouse)**	73/4228	1.05E-04	**PPARG-20887899 (mouse)**	57/3562	0.000391699
**PPARD-21283829 (human)**	65/3446	2.12E-05	**PRDM14-21183938 (mouse)**	43/1943	1.79866E-06
**PPARG-20176806 (mouse)**	16/837	0.028046641	**RAD21-21589869 (mouse)**	47/2034	1.69213E-07
**PPARG-20887899 (mouse)**	57/3562	0.003648347	**RARG-19884340 (mouse)**	18/380	1.40367E-07
**PRDM14-21183938 (mouse)**	46/1943	1.76E-06	**RCOR3-21632747 (mouse)**	50/2851	0.000116647
**RAD21-21589869 (mouse)**	52/2034	3.31E-08	**REST-18959480 (mouse)**	40/2866	0.02523314
**RCOR1-19997604 (mouse)**	42/2376	0.002348902	**REST-21632747 (mouse)**	42/2339	0.000266156
**RCOR3-21632747 (mouse)**	68/2851	2.88E-09	**RNF2-16625203 (mouse)**	48/1216	2.54134E-15
**REST-18959480 (mouse)**	69/2866	1.47E-09	**RNF2-18974828 (mouse)**	41/1298	2.58996E-10

**REST-19997604 (mouse)**	34/2118	0.02220041	**RUNX1-17652178 (human)**	18/1002	0.015251868
**REST-21632747 (mouse)**	79/2339	1.72E-18	**RUNX1-20887958 (mouse)**	25/1120	0.000254168
**RNF2-16625203 (mouse)**	58/1216	4.53E-20	**SALL4-18804426_ESC (mouse)**	21/1065	0.003371447
**RNF2-18974828 (mouse)**	63/1298	3.78E-22	**SCL-21571218 (human)**	29/1783	0.009105407
**RUNX1-17652178 (human)**	22/1002	0.002411135	**SETDB1-19884257 (mouse)**	53/2353	5.54684E-08
**SALL4-18804426_ESC (mouse)**	20/1065	0.017997901	**SIN3A-21632747 (mouse)**	19/1186	0.035576627
**SCL-21571218 (human)**	29/1783	0.028710548	**SIN3B-21632747 (mouse)**	57/4302	0.021003191
**SETDB1-19884255 (mouse)**	38/2019	0.001315722	**SMAD1-18555785 (mouse)**	12/610	0.023917966
**SETDB1-19884257 (mouse)**	42/2353	0.001972986	**SMAD-19615063 (human)**	4/124	0.04010769
**SIN3B-21632747 (mouse)**	85/4302	1.16E-07	**SMAD2-18955504 (human)**	31/1933	0.008589601
**SMAD1-18555785 (mouse)**	16/610	0.001732068	**SMAD3-18955504 (human)**	31/1933	0.008589601
**SMAD3-21741376 (human)**	32/1458	2.72E-04	**SMAD3-21741376 (human)**	22/1458	0.043313427
**SMAD4-21741376 (human)**	64/2733	1.81E-08	**SMAD4-21741376 (human)**	40/2733	0.012781268
**SOX17-20123909 (mouse)**	32/1946	0.019471536	**SOX2-16153702 (human)**	27/1278	0.000337748
**SOX2-18358816 (mouse)**	20/785	6.88E-04	**SOX2-18358816 (mouse)**	16/785	0.007377331
**SOX2-18555785 (mouse)**	15/497	6.25E-04	**SOX2-18692474 (mouse)**	71/3316	1.54968E-09
**SOX2-18692474 (mouse)**	59/3316	2.54E-04	**SOX2-20726797 (human)**	52/2561	1.65688E-06
**SOX2-19030024 (mouse)**	22/863	3.74E-04	**SOX2-21211035 (human)**	54/3413	0.000736721
**SOX2-20726797 (human)**	39/2561	0.030656162	**SPI1-20517297 (human)**	23/1248	0.00491493
**SOX2-21211035 (human)**	98/3413	2.52E-18	**STAT3-19079543 (mouse)**	21/948	0.000861265
**SPI1-20517297 (human)**	21/1248	0.042757293	**STAT4-19710469 (mouse)**	41/1933	8.67077E-06
**STAT3-19079543 (mouse)**	22/948	0.001236267	**SUZ12-16625203 (mouse)**	44/1266	2.64728E-12
**SUZ12-16625203 (mouse)**	64/1266	2.18E-23	**SUZ12-18555785 (mouse)**	36/1058	5.19214E-10
**SUZ12-18555785 (mouse)**	56/1058	2.22E-21	**SUZ12-18692474 (mouse)**	60/1905	1.10494E-14
**SUZ12-18692474 (mouse)**	102/1905	5.97E-40	**SUZ12-18974828 (mouse)**	63/1929	4.10846E-16
**SUZ12-18974828 (mouse)**	90/1929	1.13E-30	**SUZ12-20075857 (mouse)**	125/4353	3.37801E-28
**SUZ12-20075857 (mouse)**	170/4353	4.54E-52	**TAL1-20566737 (mouse)**	42/1875	1.78217E-06
**TBX3-20139965 (mouse)**	25/1068	5.21E-04	**TBX3-20139965 (mouse)**	17/1068	0.047365839
**TCF3-18347094 (mouse)**	48/2217	1.08E-05	**TCF3-18347094 (mouse)**	42/2217	8.63444E-05
**TCF3-18467660 (mouse)**	29/1388	0.001107459	**TCF3-18692474 (mouse)**	35/1351	6.08616E-07
**TCF3-18692474 (mouse)**	29/1351	7.39E-04	**TFAP2A-17053090 (human)**	36/1902	0.000288408
**TCFCP2L1-18555785 (mouse)**	41/1987	1.38E-04	**TP53-18474530 (human)**	23/826	1.87183E-05
**TET1-21451524 (mouse)**	29/1839	0.04007754	**TP53-20018659 (mouse)**	39/1122	5.11982E-11
**TFAP2A-17053090 (human)**	35/1902	0.002904483	**TP63-19390658 (human)**	5/179	0.038260634
**TFAP2C-20629094 (human)**	24/1203	0.005076762	**WT1-20215353 (mouse)**	32/1663	0.000480169
**TP53-18474530 (human)**	21/826	5.23E-04	**YAP1-20516196 (mouse)**	50/2326	5.44379E-07
**TP53-20018659 (mouse)**	38/1122	2.74E-09	**ZFP281-18358816 (mouse)**	11/578	0.036364412
**WT1-19549856 (human)**	7/199	0.008076869	**ZFP281-18757296 (mouse)**	34/2000	0.002510068
**YAP1-20516196 (mouse)**	57/2326	2.77E-08	**ZIC3-20872845 (mouse)**	8/365	0.03522598
**ZFP281-18358816 (mouse)**	12/578	0.031515259			
**ZFP281-18757296 (mouse)**	37/2000	0.002031044			
**ZIC3-20872845 (mouse)**	11/365	0.003240347			

***Listed in alphabetical order**

Supplemental References

1. Liu X, Ma L, Li HH, Huang B, Li YX, Tao YZ. beta-Arrestin-biased signaling mediates memory reconsolidation. *Proc Natl Acad Sci U S A* **112**, 4483-4488 (2015).

2. Li H, Tao Y, Ma L, Liu X. beta-Arrestin-2 inhibits preference for alcohol in mice and suppresses Akt signaling in the dorsal striatum. *Neurosci Bull* **29**, 531-540 (2013).

S1

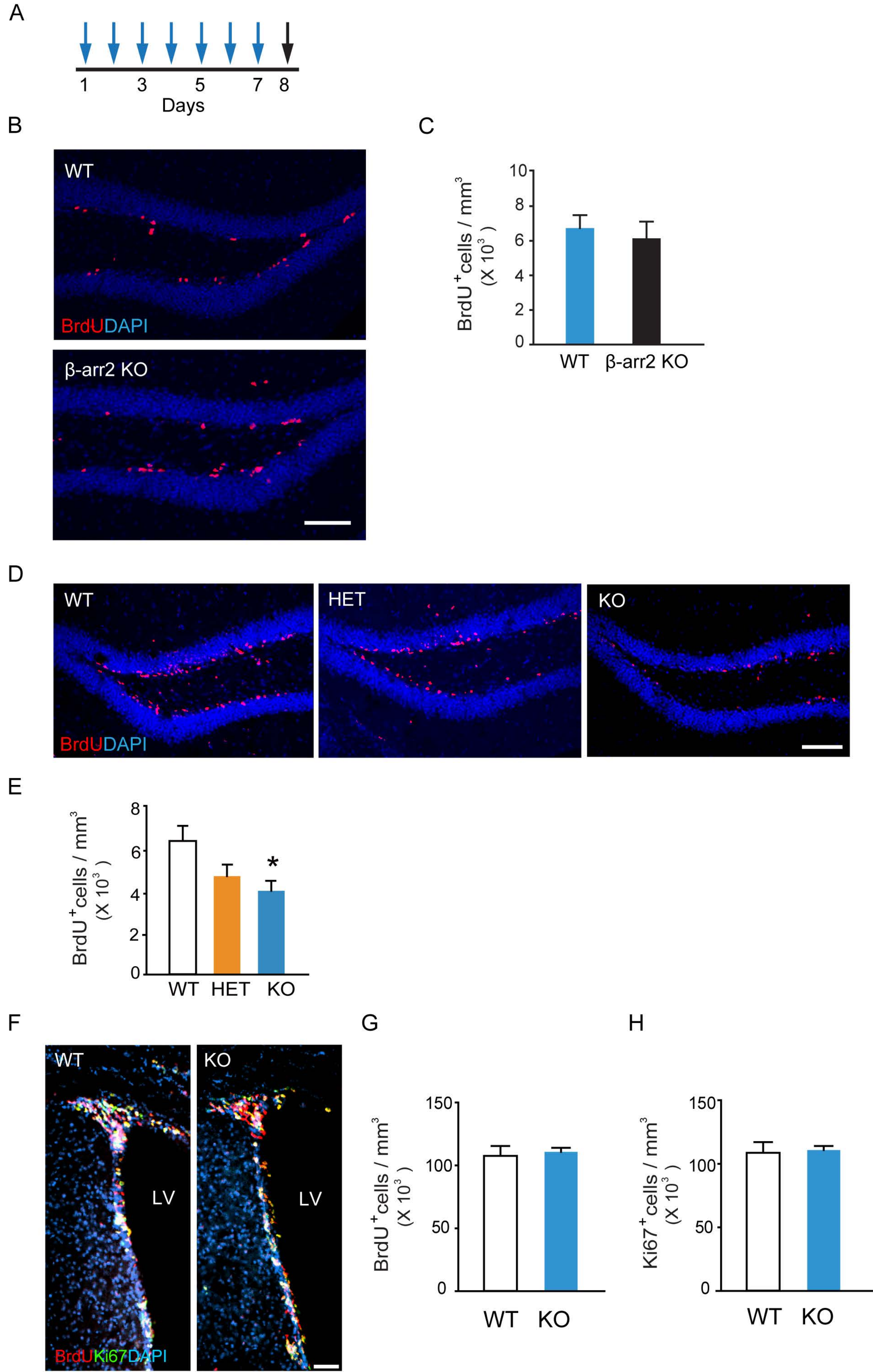
A
1
3
5
7
8
Days
B
WT
BrdUDAPI
β-arr2 KO
C
BrdU+cells / mm3 (X 10³)
10
8
6
4
2
0
WT
β-arr2 KO
D
WT
HET
KO
BrdUDAPI
E
BrdU+cells / mm3 (X 10³)
8
6
4
2
0
*
WT
HET
KO
F
WT
KO
LV
LV
BrdUKi67DAPI
G
BrdU+cells / mm3 (X 10³)
150
100
50
0
WT
KO
H
Ki67+cells / mm3 (X 10³)
150
100
50
0
WT
KO

S2

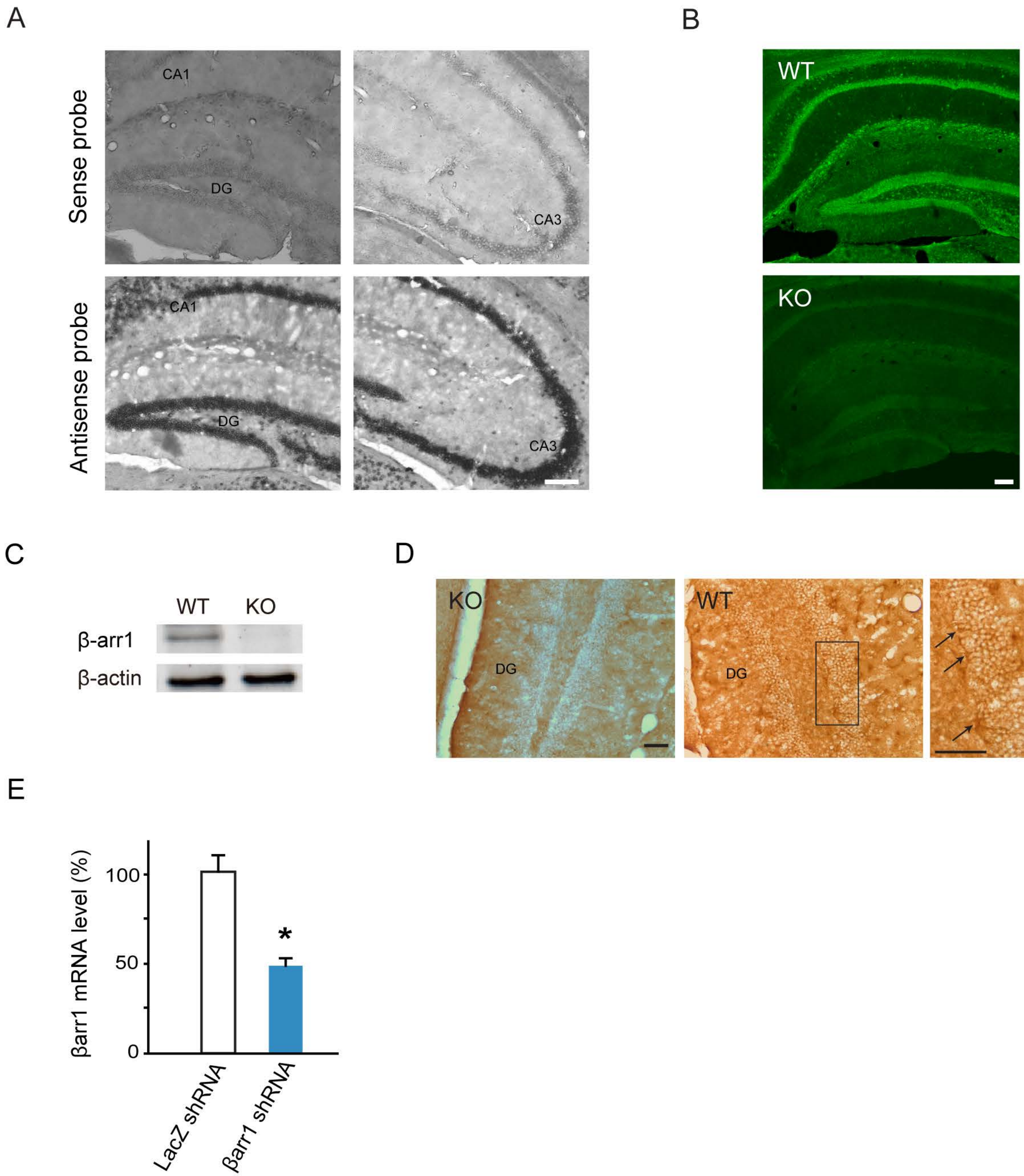

A
Sense probe
Antisense probe
CA1
DG
CA3
CA1
DG
CA3
B
WT
KO
C
WT
KO
β-arr1
β-actin
D
KO
DG
WT
DG
E
βarr1 mRNA level (%)
100
50
0
*
LacZ shRNA
βarr1 shRNA

S3

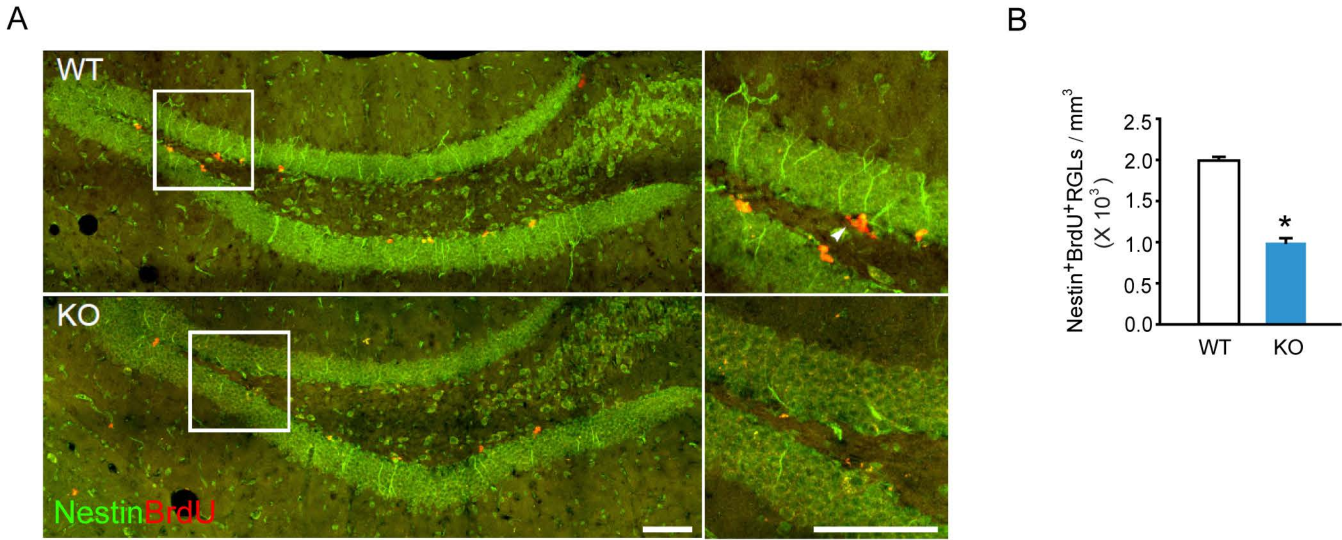
A
WT
KO
NestinBrdU
B
Nestin⁺BrdU⁺RGLs / mm³ (X 10³)
2.5
2.0
1.5
1.0
0.5
0.0
*
WT
KO

S4

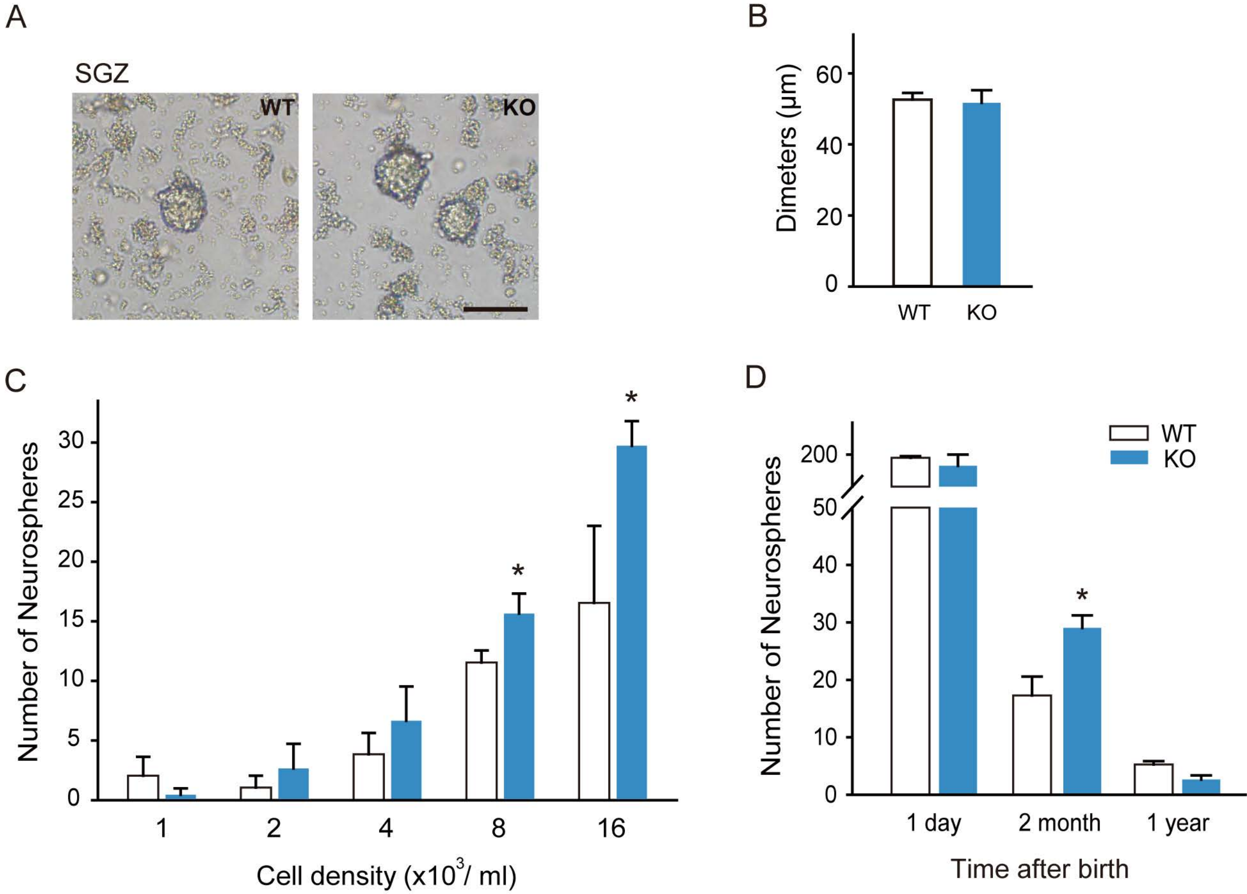
A
SGZ
WT
KO
B
Dimeters (μm)
60
40
20
0
WT
KO
C
Number of Neurospheres
30
25
20
15
10
5
0
*
*
1
2
4
8
16
Cell density (x10³/ ml)
D
Number of Neurospheres
WT
KO
200
50
40
30
20
10
0
*
1 day
2 month
1 year
Time after birth

S5

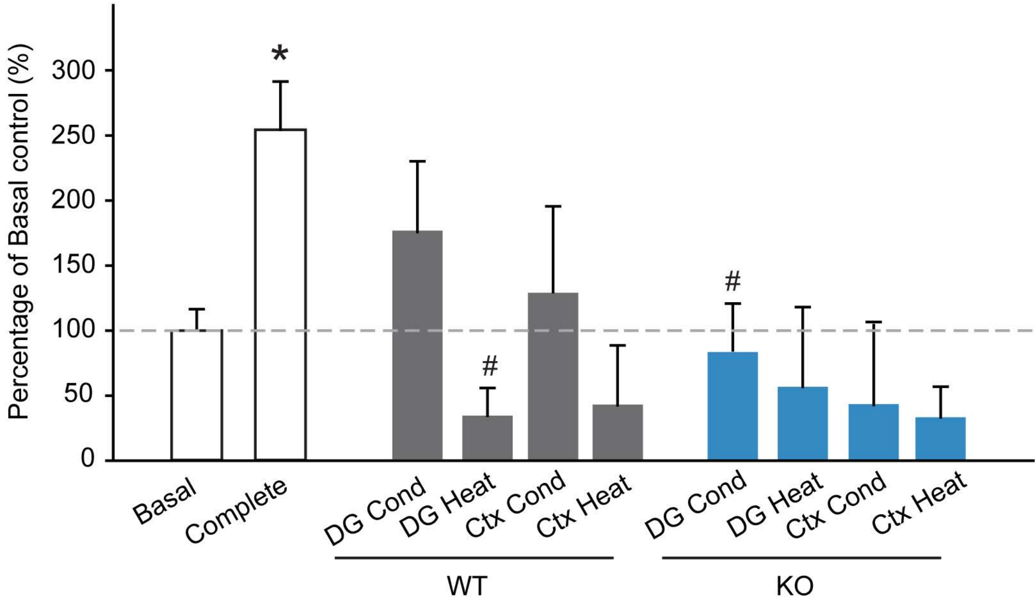
Percentage of Basal control (%)
0
50
100
150
200
250
300
*
#
#
Basal
Complete
DG Cond
DG Heat
Ctx Cond
Ctx Heat
DG Cond
DG Heat
Ctx Cond
Ctx Heat
WT
KO

S6

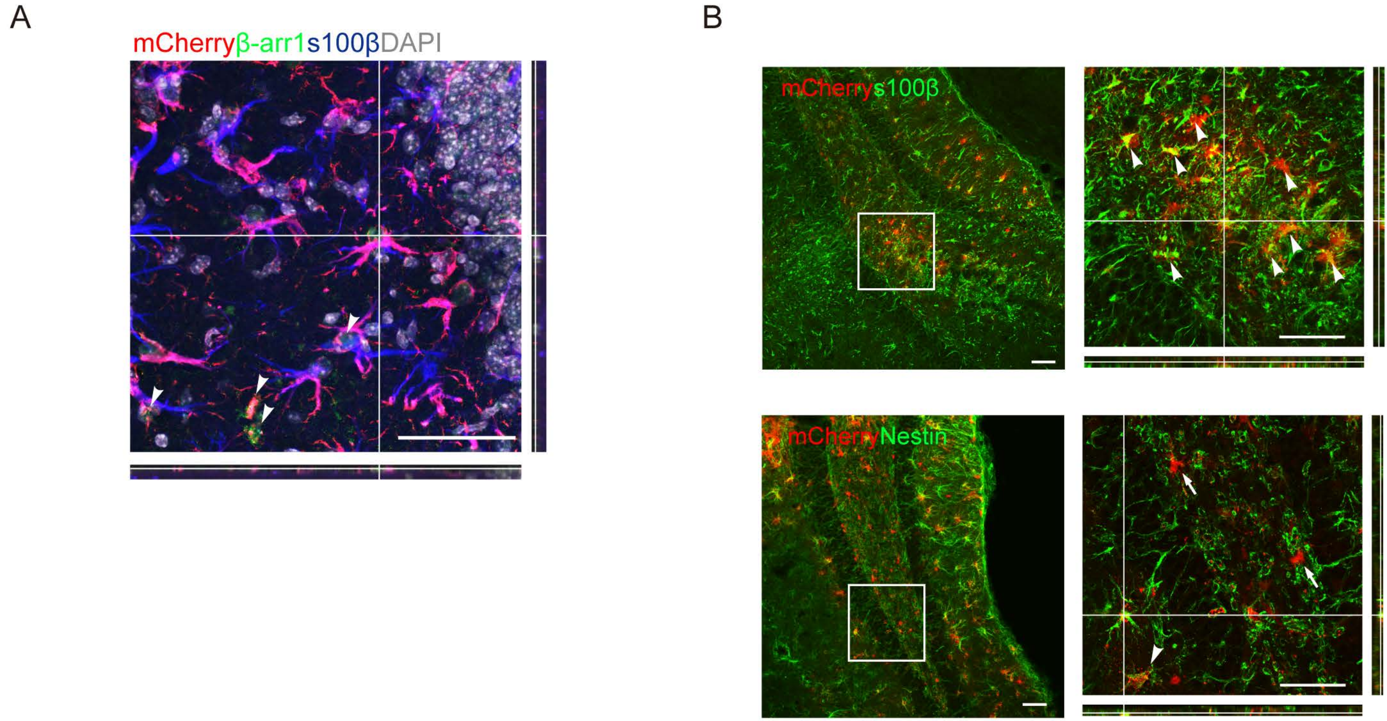

S7

A

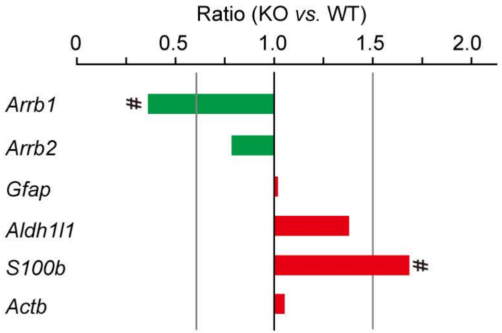

Ratio (KO *vs.* WT)
0
0.5
1.0
1.5
2.0
Arrb1
#
Arrb2
Gfap
Aldh1l1
S100b
#
Actb

B

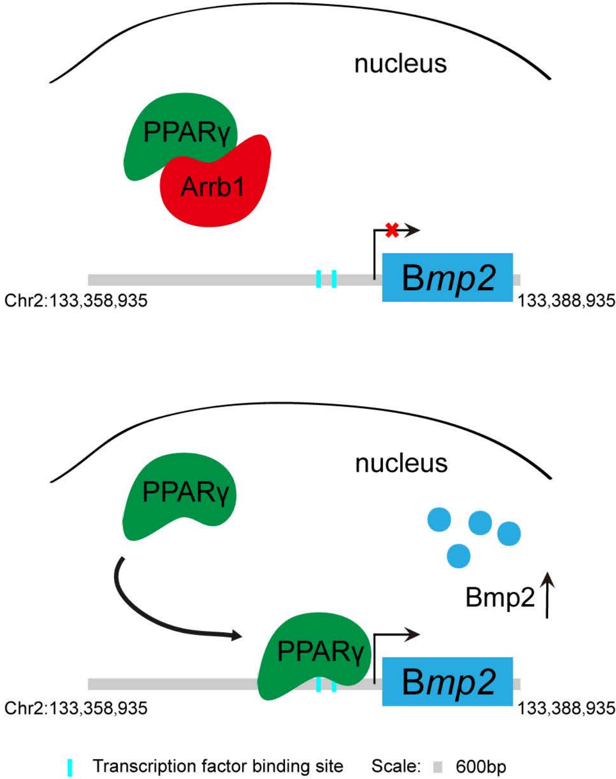

nucleus
PPARγ
Arrb1
B*mp2*
Chr2:133,358,935
133,388,935
nucleus
PPARγ
PPARγ
Bmp2
B*mp2*
Chr2:133,358,935
133,388,935
Transcription factor binding site
Scale:
600bp
